# Think Beyond The Core: Computationally Decoding the Hydrophilic Corona of Drug‐Loaded Polymer Micelles

**DOI:** 10.1002/smll.74919

**Published:** 2026-08-08

**Authors:** Maksym Karachevtsev, Josef Kehrein, Terttu Hukka, Alex Bunker, Mikko Karttunen, Robert Luxenhofer

**Affiliations:** ^1^ Drug Research Program, Division of Pharmaceutical Biosciences Faculty of Pharmacy University of Helsinki Helsinki Finland; ^2^ Soft Matter Chemistry, Department of Chemistry, Faculty of Science University of Helsinki Helsinki Finland; ^3^ Institute for Pharmacy and Food Chemistry University of Würzburg, Am Hubland Würzburg Germany; ^4^ Faculty of Engineering and Natural Sciences Materials Science and Environmental Engineering Chemistry & Advanced Materials, Tampere University Tampere Finland; ^5^ European Laboratory for Learning and Intelligent Systems (ELLIS) Institute Finland Espoo Finland; ^6^ Department of Technical Physics University of Eastern Finland Kuopio Finland

**Keywords:** copolymer, hydrogen bond, molecular dynamics, nanoformulations, polymer, sarcosine

## Abstract

Polymeric micelles are established delivery platforms for hydrophobic drugs. The molecular interactions governing their structure and function remain, however, poorly understood. All‐atom molecular dynamics simulations are used to investigate drug‐loaded ABA‐type triblock copolymer micelles with a hydrophobic poly(2‐*n*‐butyl‐2‐oxazine) core and hydrophilic coronas composed of poly(ethylene glycol) (pEG), poly(*N*,*N*‐dimethylacrylamide) (pDMAA), and poly(sarcosine) (pSAR). Here, pDMAA and pSAR are considered as alternative polymers to address emerging pEG immunogenicity. Three micellar formulations are examined at moderate (20%) and high (60%) loadings of curcumin as a model hydrophobic drug. The simulations reveal that pEG‐based micelles exhibit higher hydration and looser corona structures compared to pDMAA and pSAR micelles. In pEG micelles, curcumin localizes primarily within the hydrophobic core, whereas in pDMAA and pSAR micelles it is more uniformly distributed. At both drug loadings, curcumin preferentially aggregates into a single stable cluster, not densely packed, and interpenetrated by polymer chains. Micelles with pDMAA and pSAR coronas exhibit enhanced stability, reflecting higher internal density and tighter curcumin packing. Hydrogen bond analysis reveals the strongest curcumin‐hydrophilic A‐block interactions in pDMAA micelles, moderate loading‐dependent interactions in pSAR micelles, and minimal hydrogen‐bonding in pEG micelles due to corona hydration.

## Graphical abstract

Drug‐loaded ABA‐type triblock copolymer micelles with a hydrophobic poly(2‐n‐butyl‐2‐oxazine) core (B‐block) and hydrophilic coronas (pEG, pDMAA, pSAR (A‐block)) are investigated by all‐atom molecular dynamics simulations at 20% and 60% curcumin loadings. pDMAA and pSAR are examined as alternatives to address the pEG immunogenicity, forming compact, less hydrated coronas that enhance drug stabilization and promote more uniform drug distribution.

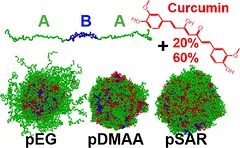

## 1 Introduction

Safe and effective delivery of poorly water‐soluble therapeutic agents remains a critical challenge in pharmaceutical research [1, 2]. To address these issues, various nanoscale drug delivery systems are being actively investigated, including liposomes, lipid nanoparticles, dendrimers, carbon‐based nanomaterials, and polymeric micelles [3, 4, 5, 6, 7, 8, 9]. The structural diversity of polymeric micelles, including telodendrimers, gradient copolymers, diblock, triblock, star‐shaped, and graft architectures, facilitates improvements in drug encapsulation efficiency and micelle stability [4, 10, 11]. Among these architectures, micelles based on amphiphilic triblock copolymers are of particular interest due to their structural versatility, high drug‐loading capacity, and biocompatibility [12, 13, 14, 15].

**FIGURE 1:**
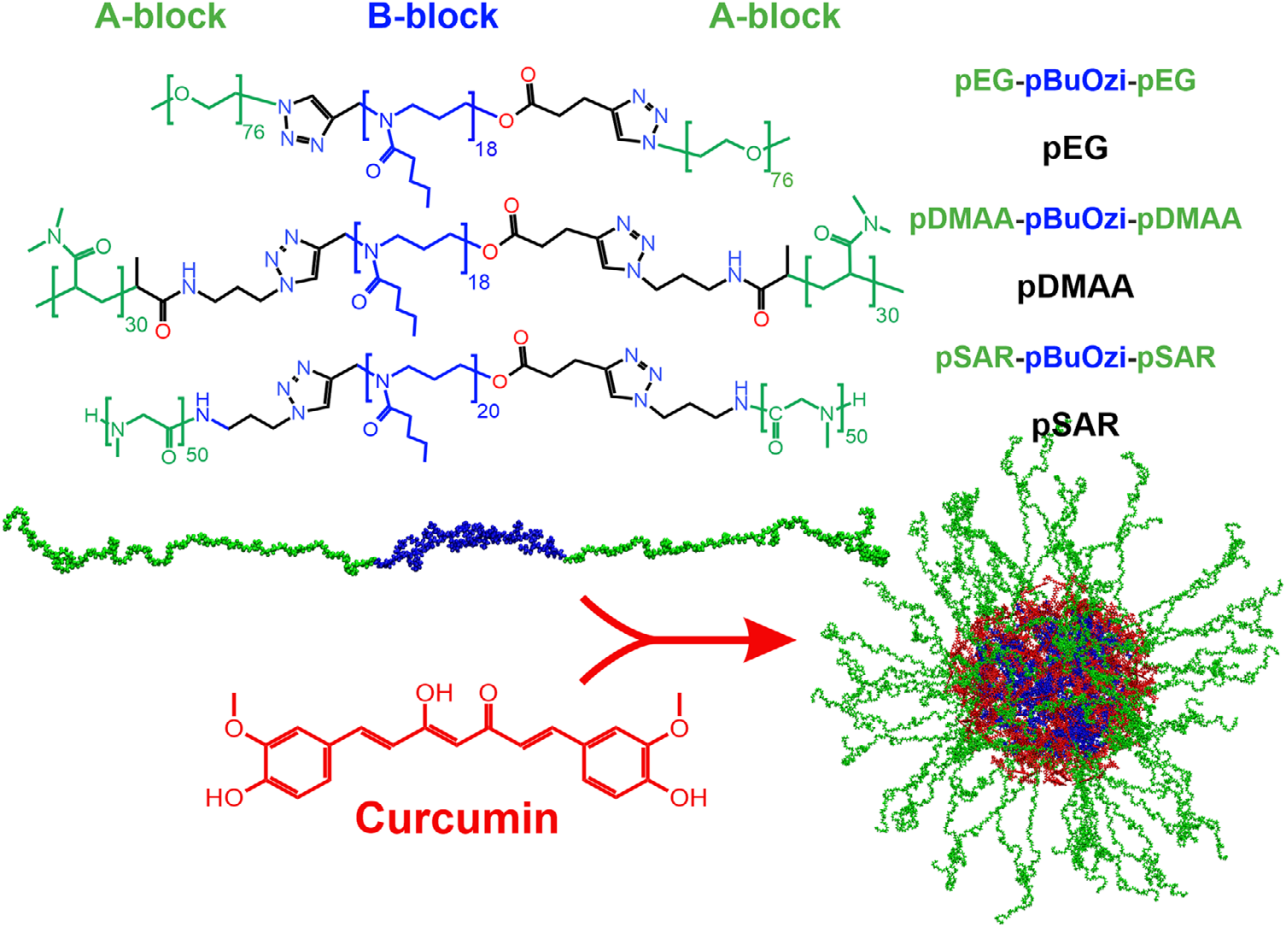
Structures of ABA‐type triblock copolymers forming micelles with a hydrophobic pBuOzi core and hydrophilic corona‐forming A‐blocks investigated in this study: pEG (76 monomers per A‐block), pDMAA (30 monomers per A‐block), and pSAR (50 monomers per A‐block). The pBuOzi B‐block length was 18 monomer units for pEG‐ and pDMAA‐based copolymers and 20 monomer units for the pSAR‐based copolymer. The hydrophilic and hydrophobic blocks are connected by a triazol linker, stemming from the highly efficient copper catalyzed 1,3‐dipolar cycloaddition between an azide‐functionalized hydrophilic block and a hydrophobic block featuring two alkyne moieties. The structure of curcumin, a model hydrophobic drug, is shown in the bottom left, and a representative micelle conformation is illustrated on the right.

Polymeric micelles are typically formed from amphiphilic di‐ or triblock copolymers (AB or ABA architecture), featuring a hydrophilic A‐block and a hydrophobic B‐block (Figure 1). In an aqueous solution, the hydrophobic B‐blocks self‐assemble into a micellar core, providing a nanoscopic domain compatible with hydrophobic drug molecules, while the hydrophilic A‐blocks form the corona, which provides colloidal stability and prevents aggregation [14]. The most common hydrophilic polymer used for the A‐blocks is poly(ethylene glycol) (pEG), owing to its excellent solubility in water and organic solvents, flexibility, and ability to impart stealth properties to nanoscale drug carriers [16, 17]. However, despite its widespread use, concerns have emerged about its immunogenicity and potential to induce hypersensitivity reactions [18, 19, 20, 21]. The extensive use of pEG in consumer products has led to a substantial portion of the population developing anti‐pEG antibodies; the prevalence of anti‐pEG antibodies has increased significantly over the past few decades: from 0.2% in the 1980s, to 25% in 2012, 44% in 2016, and to 65% in 2021 [22, 23]. Consequently, the search for alternatives to pEG that mitigate these immunogenicity issues is an active field of research [24, 25]. Promising candidates include poly(2‐oxazoline)s and poly(2‐oxazine)s, poly(sarcosine) (pSAR) and poly(*N*,*N*‐dimethylacrylamide) (pDMAA), all of which demonstrating excellent hydrophilicity and stealth behavior in biological environments [26, 27, 28]. However, pEG has very specific properties that make it a unique polymer; it is highly soluble in both polar and non‐polar solvents  [29] and is extremely flexible. Thus, comparison of the behavior of the new polymers against pEG, in the context of their current use, is needed to elucidate all implications of its replacement.

The nonionic polypeptoid pSAR exhibits physicochemical characteristics similar to pEG, including high water solubility, negligible immunogenicity, and lack of cytotoxicity, positioning it as a promising alternative [11, 27, 30, 31, 32, 33]. Molecular dynamics (MD) simulations indicate that pSAR interacts with human serum albumin with a binding profile closely resembling that of pEG, specifically in terms of affinity for specific amino acids and interaction strength [34]. Similarly, pDMAA is a hydrophilic, non‐ionic polymer with a strong affinity for water molecules [35]. ABA block copolymer micelles, specifically those based on poly(2‐oxazoline) and poly(2‐oxazine) block copolymers, exhibit exceptionally high potential for hydrophobic drug loading, achieving overall drug loadings of ∼50 wt.% with high colloidal stability [36]. Importantly, this high loading capacity is attributed to the hydrophilic corona contributing significantly to drug solubilization [37, 38, 39, 40].

In our previous work, all‐atom molecular modeling elucidated the drug‐polymer interaction mechanism within the hydrophobic core and hydrophilic corona [41], corroborating experimental evidence that both regions are crucial for achieving high drug loading [42]. A recent review of MD modeling in pharmaceutical research is provided by Mundada et al. [43]. The rational design of next‐generation stealth polymers is, however, hampered by a lack of fundamental understanding of how the hydrophilic corona interacts with both the drug and its environment at the molecular level. A direct, comparative analysis of leading pEG alternatives within the same high‐performance micellar system is critically needed to elucidate these structure‐property relationships and enable the development of superior drug delivery vehicles. To address this gap, we investigate the role of the hydrophilic corona in drug‐solubilizing micelles by designing systems with varied hydrophilic A‐blocks (pEG, pDMAA, and pSAR), while retaining the high‐performance hydrophobic poly(2‐n‐butyl‐2‐oxazine) (pBuOzi) B‐block. We employ all‐atom MD simulations to systematically investigate the structural and dynamic properties of these micelles loaded with curcumin, a model hydrophobic drug. This natural compound exhibits a broad spectrum of pharmacological activities [44, 45], but its utility is complicated by its classification as a pan‐assay interfering substance [46, 47]. Three polymer micelle systems were examined at two polymer/drug mass ratios (20% and 60%) to analyze their morphology, hydration patterns, hydrogen bonding, drug‐polymer interactions, and drug mobility, with a focus on the higher drug loading of 60%.

## 2 Methods

The RESP ESP charge Derive Server [48, 49], in combination with the Gaussian software package [50] (Hartree–Fock/6‐31G(d) theory level), was used to derive partial charges for polymers and curcumin. Atom types and bonded parameters were adapted from the Amberff14SB force field [51]. For the atoms in the pEG chains, the refined partial charges developed by Sherck et al. [52] were employed, as they more accurately reproduce the structural properties of pEG.

To maintain consistency with our previous studies [41], the hydrophilic A‐blocks were chosen to have equal molecular weights. However, we note that this design choice introduces a variable: because pEG has a lower mass per repeat unit, each A‐block (76 monomers) is longer than the coresponding A‐blocks of pSAR (50 monomers) and pDMAA (30 monomers). Matching molecular weights was selected, as nanomedicine applications typically rely on blocks of equal mass to achieve comparable hydrodynamic sizes and maintain control over the hydrophilic–lipophilic balance [53]. Alternative A‐block design strategies, such as matching the degree of polymerization or corona volume fractions, would lead to substantial differences in corona mass density or require iterative adjustment of chain length, grafting density (aggregation number), and core size. This difference in corona thickness, combined with the variation in chemical properties, is expected to influence micelle structure and drug solubilization, providing an opportunity to de‐convolute the effects of chemical composition from those of physical dimensions of the hydrophilic corona. The hydrophobic pBuOzi B‐block consisted of 18 monomer units in the PEG‐ and pDMAA‐based copolymers and 20 monomer units in the pSAR‐based copolymer. This minor variation reflects the selection of polymer compositions that have been previously synthesized and experimentally characterized [36, 37, 38, 39, 40]. The free energy of transfer of a single monomer from water to the hydrophobic core is estimated to be 1‐2 $k_{B}T$ [24]. Thus, a difference of two monomer units corresponds to a stabilization of 2‐4 $k_{B}T$ and can be regarded as a modest perturbation relative to the corona‐driven effects described herein.

The initial polymer structures were built using tleap from AmberTools22 [54]. Each polymer strand was energy‐minimized and subsequently equilibrated for 400 ps using the Generalized Born implicit solvent model [55] with sander from AmberTools22. To construct the micelle, 35 polymer strands were arranged into a spherical configuration using packmol [56], with the hydrophobic B‐blocks positioned in the center of the micelle, and the hydrophilic A‐blocks directed away from the micelle core, resulting in micelles with an approximate the radius of gyration ($R_{g}$) 6.3–11.6 nm. Curcumin molecules were inserted at two different loadings: 20% and 60% (w/w relative to polymer) so that 50% were randomly placed within the hydrophobic B‐block region and the remaining 50% within the hydrophilic A‐block region. We employed 35 polymer strands per micelle to balance realistic core volume with computational feasibility. Two independent replicas with new placements were generated for each micelle composition and curcumin concentration, resulting in a total of 12 simulated systems. The simulation details for all systems, including the number of curcumin molecules, final box size, equilibration duration, and production run length, are summarized in Table S1.

Each micelle was solvated in a cubic water box of TIP3P water molecules [57] with a minimum polymer‐to‐border distance of 1 nm. For pEG micelles, the minimum polymer‐to‐border distance was 3.2 nm to account for the extended length and high flexibility of the pEG polymer, and to ensure accurate treatment of periodic boundary conditions. Chloride ions (70 in total) were added to neutralize the charge of the pSAR polymers.

The MD simulations were carried out using GROMACS 2022.4 [58]. Each system underwent energy minimization, followed by a 100 ps (2 fs time step) NVT pre‐equilibration at 300 K using the velocity‐rescale thermostat of Bussi et al. [59] with a coupling constant of 0.1 ${\mathrm{ps}}^{-1}$. Subsequently, a 20 ns NPT equilibration run was performed with a 2 fs time step. Pressure was maintained using the Parrinello–Rahman barostat [60] with a reference pressure of 1.0 bar and a coupling constant of $4.5\times 10^{-5}$ ${\mathrm{bar}}^{-1}$, and a barostat time constant of 2 ps. The Particle–Mesh Ewald [61, 62] method was used to account for electrostatic interactions. To accelerate polymer chain reorganization and promote rapid micelle self‐assembly, the system was rapidly heated from 300 to 385 K within the first 800 ps of the simulation. This elevated temperature facilitates exploration of the conformational space while remaining below the degradation temperature of the polymers. During this process, extended polymer chains condensed onto the hydrophobic micelle core.

After the NPT equilibration, the micelles with the 1 nm water layer were removed from the water box and re‐solvated in a new box to decrease the system size. The systems were then resolvated in new water boxes with a minimum polymer‐to‐border distance of 1 nm for further simulation. For the pEG micelles, the minimum polymer‐to‐box edge distance was 3.2 nm. An NPT equilibration was carried out at 385 K, with rapid heating applied during the first 800 ps. All other simulation parameters were kept unchanged. After equilibration, a 400 ns production run was conducted, with the system cooled to 300 K during the first 800 ps.

All trajectory analyzes were performed using cpptraj from AmberTools22 [54] and MDAnalysis [63, 64] HB lifetimes were calculated using MDAnalysis, applying distance cutoffs determined from the first minima in the corresponding radial distribution function (RDF) plots. The time correlation function for HBs, C(τ), was computed using

(1) $$C\left(\tau \right)=\langle \frac{h_{ij}\left(t_{0}\right)\;h_{ij}\left(t_{0}+\tau \right)}{h_{ij}^{2}\left(t_{0}\right)}\rangle ,$$

where $h_{ij}\left(t_{0}\right)$ and $h_{ij}\left(t_{0}+\tau \right)$ are equal to 1 if a HB between atoms i and j exists at times $t_{0}$ and $t_{0}+\tau$, and 0 otherwise  [65, 66].

To extract characteristic lifetimes, autocorrelation curves were fitted with a biexponential function:

(2) $$C\left(t\right)=a\;e^{-t/\tau_{1}}+b\;e^{-t/\tau_{2}},$$

where $\tau_{1}$ and $\tau_{2}$ represent two characteristic time constants corresponding to processes occurring on different timescales: $\tau_{1}$ describes a short‐timescale (fast) process, while $\tau_{2}$ corresponds to a longer‐timescale (slow) process. The coefficients a and b sum to 1 and represent their relative contributions The overall characteristic hydrogen bond lifetime $\tau_{\mathrm{HB}}$ was then obtained by integrating the fitted autocorrelation function analytically [67, 68]. Cluster analysis of curcumin–curcumin interactions was performed using the gmx_cluster utility implemented in GROMACS [58]. The analysis was based on pairwise distances between curcumin atoms, with a cutoff value of 3.5 Å used to define cluster membership.

The cone algorithm [69] was employed for analysis of the surface exposure of the curcumin atoms. A cosine value of 0.5 for the cone angle and a cone slant height of 0.5 nm were employed. Based on this analysis, the number of surface‐exposed curcumin atoms and the fraction of micelle surface atoms corresponding to the drug molecules were determined.

For all cases, the asphericity of the entire micelle, including both the hydrophobic core and corona blocks, was calculated with RDKit 2023.03.2 [70] using the last 100 ns of the production run. The asphericity parameter, Q, quantifies the deviation of the micelle from perfect sphericity and was calculated as

(3) $$Q=\frac{{\left(\lambda_{3}-\lambda_{2}\right)}^{2}+{\left(\lambda_{3}-\lambda_{1}\right)}^{2}+{\left(\lambda_{2}-\lambda_{1}\right)}^{2}}{2\left(\lambda_{1}^{2}+\lambda_{2}^{2}+\lambda_{3}^{2}\right)},$$

where $\lambda_{1}$, $\lambda_{2}$, and $\lambda_{3}$ represent the three eigenvalues of the gyration tensor. A value of Q=0 corresponds to a perfectly spherical micelle, while larger values indicate increasing elongation or anisotropy, allowing straightforward comparison of micelle shapes across systems and time frames  [71].

We calculated the $R_{g}$ for the entire micelles as well as the curcumin molecules within the micelles using the definition $R_{g}^{2}=\frac{1}{M}\sum_{i=1}^{N}m_{i}{\left(r_{i}-r_{\mathrm{cm}}\right)}^{2}$, where N is the number of atoms, $M=\sum_{i=1}^{N}m_{i}$ is the total mass, $m_{i}$ is the mass of atom i, $r_{i}$ is the position of atom i, and $r_{\mathrm{cm}}$ is the position of the center of mass.

Radial mass distribution profiles were calculated by binning atoms into concentric spherical shells centered at the micelle center of mass. The local density ρ(r) was obtained by normalizing the mass within each shell by its volume, while the radial mass distribution was computed as $\rho \left(r\right)\cdot 4\pi r^{2}$ and represents the mass contained in each shell. A bin width of 0.005 nm  was used, with a maximum radius of 10 nm .

## 3 Results and Discussion

### 3.1 Micelle Structures

**FIGURE 2:**
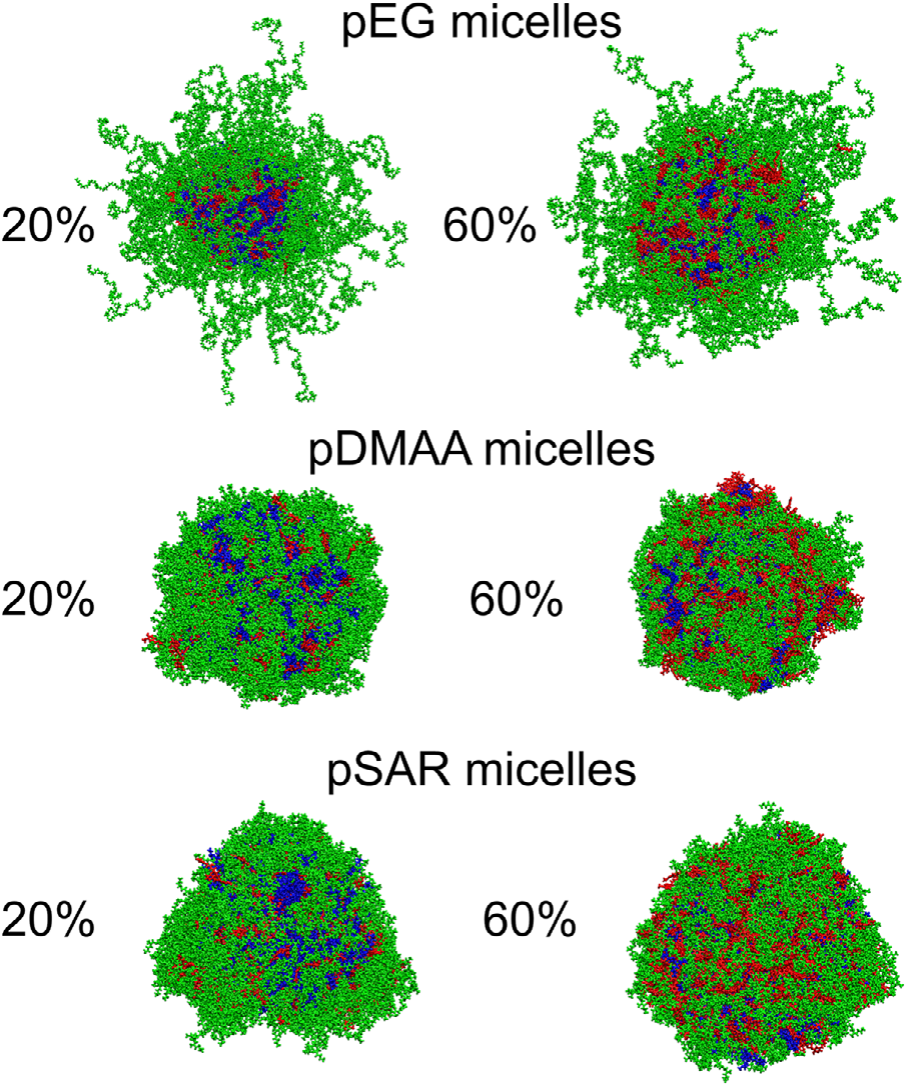
Micelle structures at 20% and 60% curcumin loading (first replica) with different hydrophilic A‐blocks (pEG, pDMAA, and pSAR) after 400 ns of production runs. Curcumin is shown in red, A‐blocks in green, and B‐blocks in blue.

During the initial phase of the simulations, the polymer chains self‐assembled into well‐defined core‐corona structures (Figure 2 and Figure S1). The time evolution of the radius of gyration ($R_{g}$) shows that the systems reach equilibrium during the equilibration run (Figure S2). Micelle morphology was quantified by calculating the asphericity parameter (Equation 3) for each system. The asphericity parameter remained low (Q<0.05, Table S2) in all systems, confirming that the micelles maintained a consistently spherical shape without significant dependence on corona chemistry or drug loading, that is, the hydrophobic core dominates micelle morphology.

**FIGURE 3:**
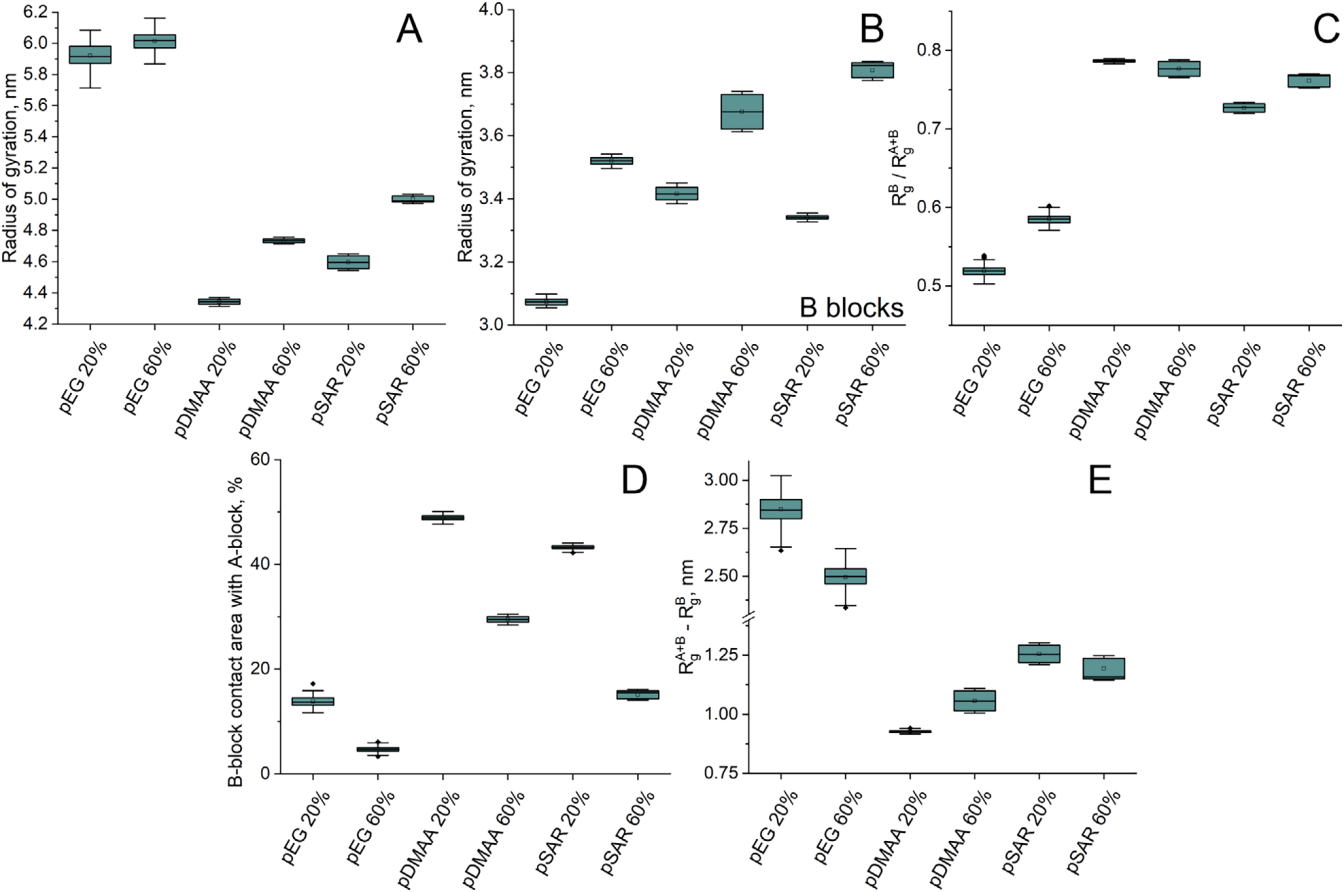
Boxplots showing the radius of gyration ($R_{g}$) of the micelle: (A) polymer atoms, (B) B‐block atoms, and (C) the ratio of the $R_{g}$ of the B‐block atoms to the $R_{g}$ of all polymer atoms, (D) percentage of B‐block contact area with A‐blocks, and (E) the difference between the $R_{g}$ of all polymer atoms and that of the B‐block, $R_{g}^{A+B}-R_{g}^{B}$.

The overall micelle size, characterized by $R_{g}$, ranged from 4.3 to 6.0 nm, and increased with higher drug loading in all systems (Figure 3A). The pEG‐based micelles were consistently the largest, reaching an $R_{g}$ of 6.0 nm at 60% drug loading. The larger size is a direct consequence of the greater flexibility and longer contour length of the pEG corona chains, which enables a more extended and hydrated corona, consistent with previous experimental and computational findings [72, 73]. The pEG polymer is extended out into the solvent resembling a random coil conformation. As an extremely flexible polymer its high solubility, in both polar and non‐polar solvents  [29] is entropically driven, thus we see the unique properties of pEG displayed. In contrast, the pSAR and pDMAA micelles exhibited smaller $R_{g}$ values (4.3–5.0 nm), comparable to those reported for micelles with poly(2‐methyl‐2‐oxazoline) (MeOx) A‐blocks [41].

Differences in the hydrophobic B‐block were observed across the three corona chemistries. At 60% drug loading, the B‐block $R_{g}$ was 3.5 nm for pEG, compared to 3.7 nm for pDMAA and 3.8 nm for pSAR, indicating that the core was most compact in pEG‐based micelles. For all systems, the B‐block $R_{g}$ increased with drug loading (Figure 3B), from 3.1–3.4 nm at 20% to 3.5–3.8 nm at 60%, reflecting the expansion of the hydrophobic core to accommodate more curcumin molecules.

In clear contrast to the B‐blocks, the pEG overall micelle size showed no statistically significant response to increased drug loading, with $R_{g}$ remaining within 5.9–6.0 nm between 20% and 60% loading (p>0.05). This was not observed for pSAR or pDMAA systems, where the corona $R_{g}$ increased modestly with drug loading. These results suggest that the highly extended and solvated pEG corona maintains its structure independently of the underlying core state.

To further characterize the relative compactness of the hydrophobic core vs. the hydrophilic corona, we calculated the ratio of all polymer atoms ($R_{g}^{B}/R_{g}^{A+B}$). This ratio was significantly lower for pEG‐based micelles ($R_{g}^{B}/R_{g}^{A+B}$ = 0.5–0.6, Figure 3C) compared to pSAR and pDMAA systems (0.7–0.8), reflecting the greater contour length of the pEG A‐block. Because of our molecular‐weight‐equivalent design strategy, the pEG A‐block is approximately 52% and 153% longer in terms of repeat units than the pSAR and pDMAA A‐blocks, respectively. This naturally results in a more extended corona that reduces the relative size of the hydrophobic core. Consistently, the difference between the radius of gyration of all polymer atoms and that of the B‐block, $R_{g}^{A+B}-R_{g}^{B}$ (Figure 3E), is largest for pEG systems, indicating a more extended hydrophilic corona. In contrast, smaller differences observed for pSAR and pDMAA systems reflect more compact coronas. This is consistent with the radial mass distribution profiles (Figure S3), where the pEG A‐block extends to larger distances than in pSAR and pDMAA micelles. Increasing drug loading from 20% to 60% broadens and shifts the B‐block and curcumin distributions outward.

The structural difference between pEG and the alternative coronas is further evidenced by the interfacial contact area between the hydrophobic core and the hydrophilic corona (Figure 3D). At 60% drug loading, the percentage of B‐block contact area with A‐blocks was 4.7% for pEG, compared to 29.5% for pDMAA and 15.1% for pSAR (p<0.05). This reduced interfacial contact indicates less interpenetration between the core and corona in pEG systems. The more extended and solvated pEG corona appears to form a more diffuse interface with the hydrophobic core, a structural feature that may have important implications for drug release kinetics and overall micelle stability.

To characterize the mutual interpenetration of the A‐ and B‐blocks, we analyzed the radial atomic distributions in the micelles (Figure S4). The pEG corona showed a distinctive distribution: no pEG atoms were found in the inner hydrophobic core (<3 nm), while approximately 8% of the pEG atoms were located in the outermost region (7–8 nm from the micelle center). In contrast, pDMAA and pSAR micelles showed minimal A‐block atoms in this outermost region, 0%–3%. Notably, atoms of the A‐blocks in pDMAA micelles penetrated relatively close to the center of the hydrophobic core.

Water was present throughout all micelle structures, including the inner hydrophobic core (up to 19% of all atoms in <1 nm region), and its content increased progressively toward the surface. The radial water distribution showed a clear gradient observed across all studied micelle systems with values reaching up to 19% in the inner core (<1 nm), up to 63% in the core‐corona interface (4–5 nm), up to 81% in the corona proper (5–6 nm), and up to 97% in the outermost corona (6–7 nm). This gradient reflects the increasing hydrophilicity moving outward from the hydrophobic core to the aqueous environment. The number of atoms of water molecules at the interface between the A‐ and B‐blocks is significantly higher in pEG micelles, reaching 63%–67% of all atoms present in this region of the micelle. In contrast, pDMAA and pSAR micelles show a smaller increase in the presence of water atoms, ranging from 25% to 44% of all atoms in this region. This difference is due to the tighter packing of A‐block atoms in pDMAA and pSAR micelles (38%–53%, 5‐6 nm) compared to the looser packing of A‐block atoms in pEG micelles (24%–27% of all atoms).

### 3.2 Micelle Hydration

**FIGURE 4:**
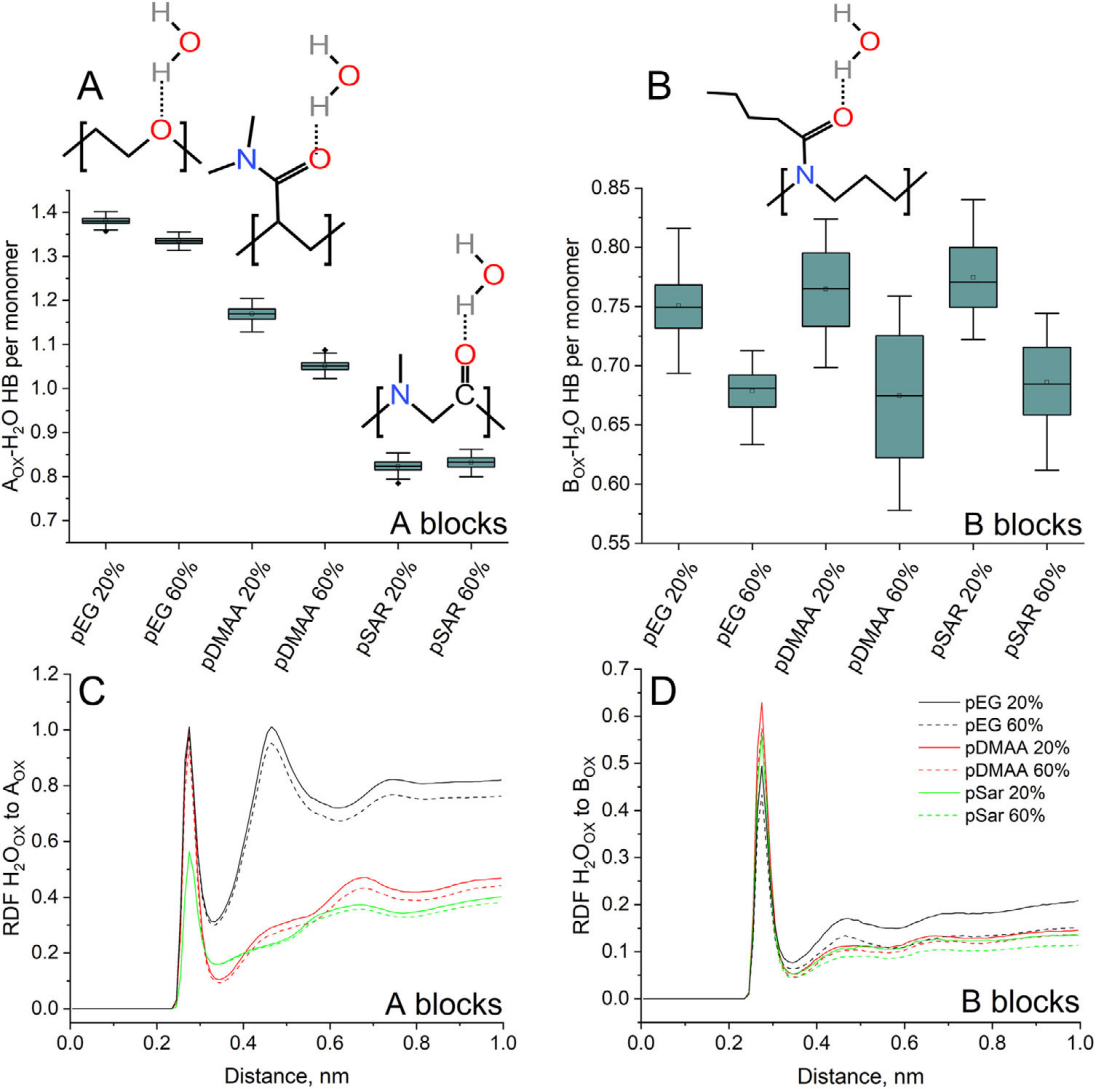
Hydration and HB analysis: Average number of HBs between water molecules and the oxygen atoms of (A) the A‐block ($A_{\mathrm{OX}}$) and (B) B‐block ($B_{\mathrm{OX}}$) polymer chains. RDF profiles for water oxygens ($H_{2}O_{\mathrm{OX}}$) found around (C) the A‐blocks and (D) B‐blocks. For each system, two replicas are shown using the same color and line style.

Micelle hydration was characterized by analyzing hydrogen bonds (HBs) between water molecules and the oxygen atoms in the hydrophilic A‐block, revealing a consistent ranking: pEG > pDMAA > pSAR (Figure 4A). At 60% drug loading, hydration levels decreased for pDMAA and pEG, but remained stable for pSAR. Furthermore, the hydration level of pDMAA was comparable to that of pMeOx and exceeded the hydration level of poly(2‐ethyl‐2‐oxazoline) (pEtOx) reported in our previous study [41]; pEtOx still forms more HBs with water than pSAR. Across both studies, pEG exhibited the highest overall hydration among all hydrophilic blocks examined.

Hydration analysis of the hydrophobic pBuOzi B‐blocks revealed significantly lower hydration levels compared to the A‐blocks. B‐block hydration was similar across all micelles and depended only on drug loading, as expected from the identical B‐block structure. The number of HBs between water and B‐blocks decreased with increasing drug loading (Figure 4B), reflecting the displacement of water by curcumin molecules competing for binding sites in the tertiary amides of the pBuOzi repeating units. Moreover and unexpectedly, the hydration level of pBuOzi is higher than that of poly(2‐propyl‐2‐oxazine) (pPrOzi) and, at 20% drug loading, is comparable to that of poly(2‐butyl‐2‐oxazoline) (pBuOx), as reported in our previous study [41].

The RDF profiles for water oxygens around the A‐blocks of the micelles (Figure 4C) revealed higher hydration levels in pEG‐based micelles compared to pDMAA and pSAR systems consistent with the coordination numbers ($N_{c}$) reported in Table S3. This difference reflects the denser packing of pDMAA and pSAR A‐blocks compared to pEG. The compact packing of pDMAA and pSAR A‐blocks increases interactions with neighboring polymer chains and curcumin molecules (see next section). In contrast, the extended pEG structure exhibits a larger $R_{g}$ and allows water molecules to penetrate deeper into the hydrophobic core, increasing their contact with water rather than with curcumin or neighboring polymer chains. The RDF profiles for the B‐blocks of all micelles are similar (Figure 4D), as expected from the identical B‐block structure.

### 3.3 Distribution of Curcumin Inside the Micelles

**FIGURE 5:**
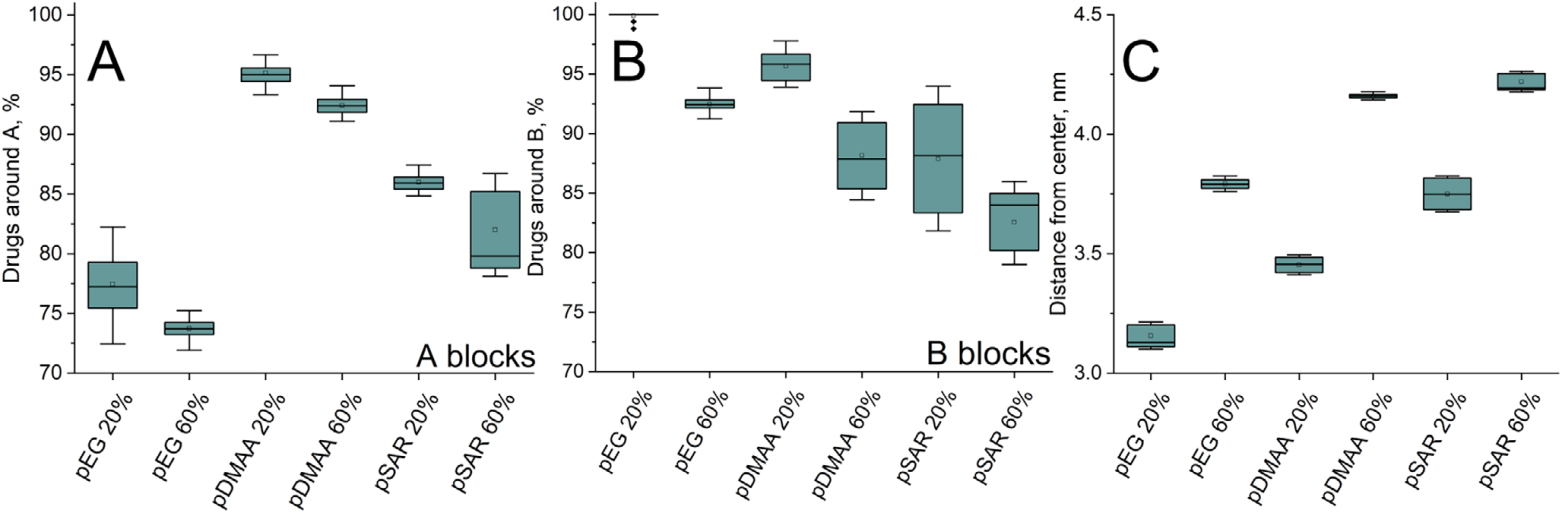
Percentage of curcumin drug molecules located at a distance of up to 0.5 nm from (A) the A‐block atoms, (B) B‐block atoms, and (C) the average distances of curcumin molecules measured from the micelle center of mass. A molecule was considered within the cutoff if at least one of its atoms was located at a distance of 0.5 nm or less.

The deeper penetration of water into pEG‐based micelles is further supported by the curcumin distribution profiles. The percentage of curcumin molecules located within 0.5 nm of A‐ and B‐blocks was analyzed (Figure 5). In addition, the number of curcumin molecules located within 0.5 nm of either the A‐ or B‐block was quantified, excluding those that were simultaneously near both blocks (Figure S5). In pEG micelles, approximately 70%–80% of curcumin molecules are positioned close to the A‐block and 92‐100% are located within or near the hydrophobic core (Figure 5B). However, only 0%–10% of curcumin molecules interact exclusively with the A‐block in pEG micelles, while 17%–22% interact solely with the B‐block (Figure S5). This localization likely reflects curcumin positioning at the A/B block interface, and the mutual penetration of A‐blocks into the hydrophobic core and B‐blocks into the hydrophilic corona, respectively.

To further characterize the internal organization of the micelles, we analyzed the radial mass density profiles of curcumin and the polymer blocks as a function of distance from the micelle center of mass (Figure S6). The profiles show that in pEG micelles, curcumin is concentrated predominantly within the B‐block‐rich core, whereas in pDMAA and pSAR micelles it is distributed more broadly toward the core‐corona interface. This analysis, together with the overlaid curcumin density profiles for all systems and replicas shown in Figure S7, indicates that in pEG micelles, curcumin is concentrated predominantly within the B‐block‐rich core, whereas in pDMAA and pSAR micelles it is distributed more broadly toward the core‐corona interface. Please note, increased fluctuations at very short radial distances arise because the spherical shell volume decreases toward the micelle center, resulting in fewer atoms contributing to the density calculation and consequently a lower signal‐to‐noise ratio.

Whereas curcumin molecules in pEG micelles are more concentrated near or within the hydrophobic B‐block, as indicated by their proximity to the micelle center (Figure 5C), curcumin in pSAR and pDMAA micelles is located further from the center at equivalent drug loading levels (Figure 5C). The pSAR and pDMAA micelles also exhibited a more uniform distribution of curcumin molecules. pDMAA micelles showed the highest percentage of curcumin near the A‐block, with more than 90% of the molecules in close proximity to this block (Figure 5A). Furthermore, 6%–10% of the curcumin molecules were located exclusively near the pDMAA A‐block (Figure S5). This distribution reflects the deeper penetration of the pDMAA A‐block into the hydrophobic core, which enables a more uniform distribution of curcumin throughout the micelle. Increasing drug loading from 20% to 60% resulted in a slight increase in the proportion of curcumin molecules in contact with only the A‐block, with a corresponding increase in their average distance from the center of the micelle (Figure 5C).

**FIGURE 6:**
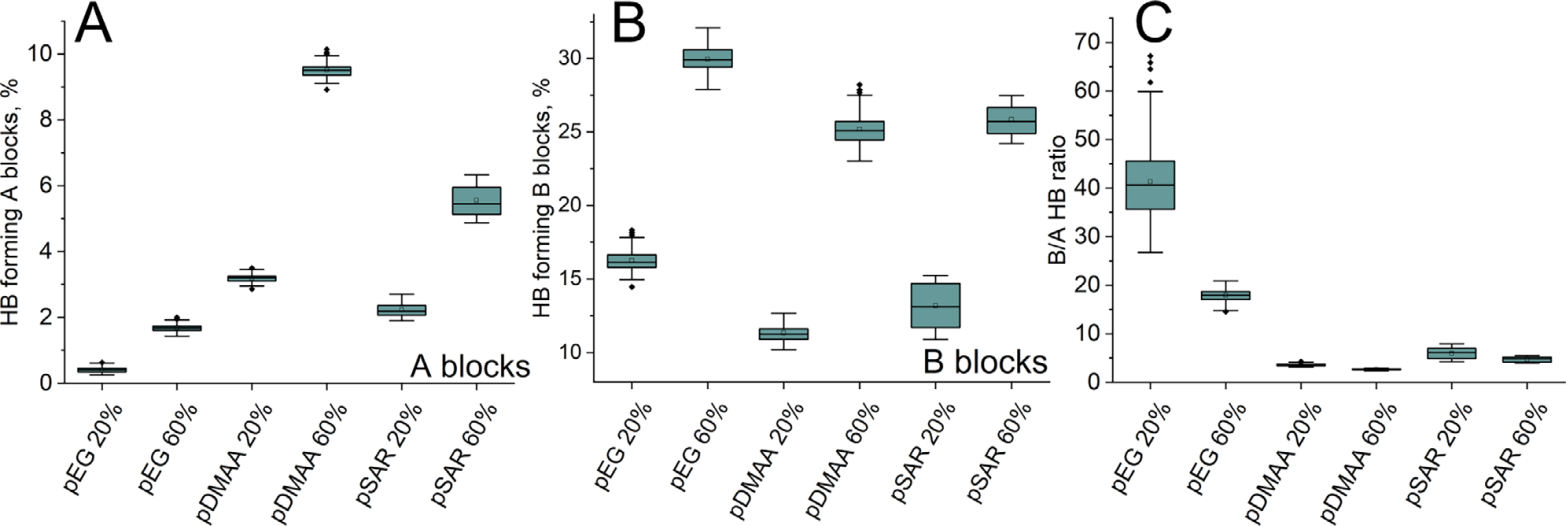
Percentage of oxygen atoms engaged in HB with curcumin molecules: (A) atoms of the A‐blocks and (B) atoms of the B‐blocks. (C) The ratio of HB occurrence between the A‐block and B‐block oxygen atoms, illustrating the relative interaction strength of curcumin with each block.

To characterize curcumin distribution and its interactions with different polymer blocks, we analyzed the percentage of oxygen atoms in the A‐ and B‐blocks that form HBs with curcumin (Figure 6). The pDMAA micelles exhibited the highest number of HBs between curcumin and A‐block atoms, particularly at 60% drug loading. In contrast, pEG micelles showed the minimum A‐block–curcumin HBs (0.3%–2%), reflecting the strong hydration of pEG chains that suppresses HB formation with curcumin. pSAR micelles exhibited a moderate number of A‐block–curcumin HBs, which increased with higher drug loading. Comparison with our previous study [41] revealed that pEG exhibited the lowest curcumin‐A‐block HBs among all polymers examined.

Most HBs formed between curcumin and the hydrophobic B‐block. Micelles containing pEG exhibited the highest percentage of B‐block–curcumin HBs (16%–30%), whereas pDMAA and pSAR micelles showed lower percentages (Figure 6). The B/A HB ratio was also highest in pEG micelles, reaching 40 at 20% drug loading, confirming the predominant localization of curcumin within the hydrophobic core (Figure 6C). At 60% drug loading, this ratio decreased to 18%, reflecting core saturation and reduced accessibility of B‐block atoms to curcumin. In contrast, pDMAA micelles showed a much lower B/A HB ratio, indicating a more uniform distribution of HBs between the B‐ and A‐blocks. Compared to previous studies, the B‐block pBuOzi formed more HBs with curcumin than the pPrOzi and pBuOx B‐blocks investigated in our previous work, at the same drug loading  [41].

**FIGURE 7:**
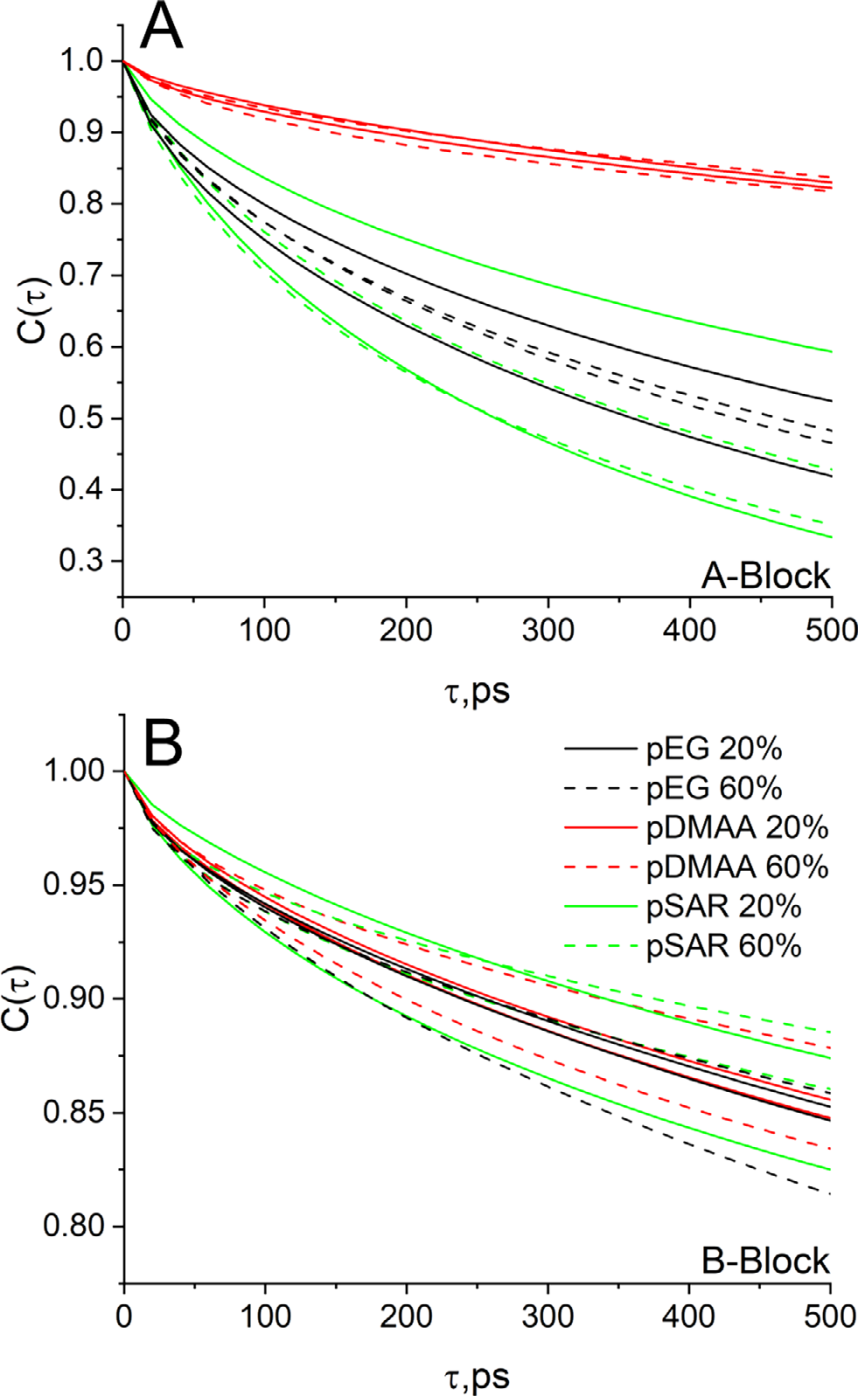
Autocorrelation functions C(τ) fitted with exponential decay curves of HBs between curcumin hydroxy groups and (A) A‐blocks and (B) B‐blocks of the micelles. Solid lines correspond to micelles containing 20% drug loading, while dashed lines represent micelles with 60% drug loading. Polymers are indicated by color: pEG (black), pDMAA (red), and pSAR (green). For each system, two replicas are shown using the same color and line style.

To further characterize these interactions, we analyzed HB lifetimes using the HB autocorrelation functions, C(τ) (Figure 7 and Table S4, which summarizes the biexponential fitting results). pDMAA micelles exhibited the longest A‐block–curcumin HB lifetimes, indicating greater bond stability. In contrast, pSAR and pEG micelles showed shorter A‐block‐curcumin HB lifetimes, which were similar to each other. B‐block–curcumin HB lifetimes were similar across all micelle types and relatively independent of drug loading, as expected from the identical B‐block structure. B‐block‐curcumin HBs were more stable (longer lifetimes) than A‐block–curcumin HBs, with the exception of pDMAA, which has similar HB lifetimes. The greater B‐block‐curcumin HB stability may influence drug release kinetics, as stronger interactions can slow the release rate and possibly enable more sustained and controlled delivery. Note that changes in HB lifetimes can affect optical properties such as fluorescence lifetimes, as demonstrated in previous studies [74].

We evaluated the distribution of curcumin on the micelle surface using a cone algorithm‐based analysis [69] (Table S5). At 20% drug loading, pDMAA micelles contained 5.2%–5.9% of curcumin atoms on the surface (4.4%–5.0% of all surface atoms). pSAR micelles contained 5.2%–7.8% of curcumin atoms at the surface (2.9%–6.5% of total surface atoms). At 60% drug loading, surface‐exposed curcumin increased substantially: pDMAA micelles reached 8.6%–9.6% of curcumin atoms at the surface (20.1%–22.1% of all surface atoms), while pSAR micelles reached 7.1%–7.9% (16.1%–18.2% of surface atoms). Notably, curcumin atoms at the micelle surface were not evenly distributed, but rather localized into distinct hydrophobic “sticky” areas, consistent with previous studies [41]. These sticky areas may affect colloidal stability, enhance binding to cell membranes or proteins, and influence endocytosis and pharmacokinetics both in vitro and in vivo. Experimental studies will be needed to show which effects prevail.

In the pEG micelles, the number of curcumin atoms detected at the surface does not reflect the actual drug presence at the micelle–water interface. Visual analysis throughout the simulation revealed that curcumin remained localized within or near the hydrophobic B‐block and was largely absent from the pEG corona. The surface data likely reflect the reduced density and increased hydration of the pEG corona.

**FIGURE 8:**
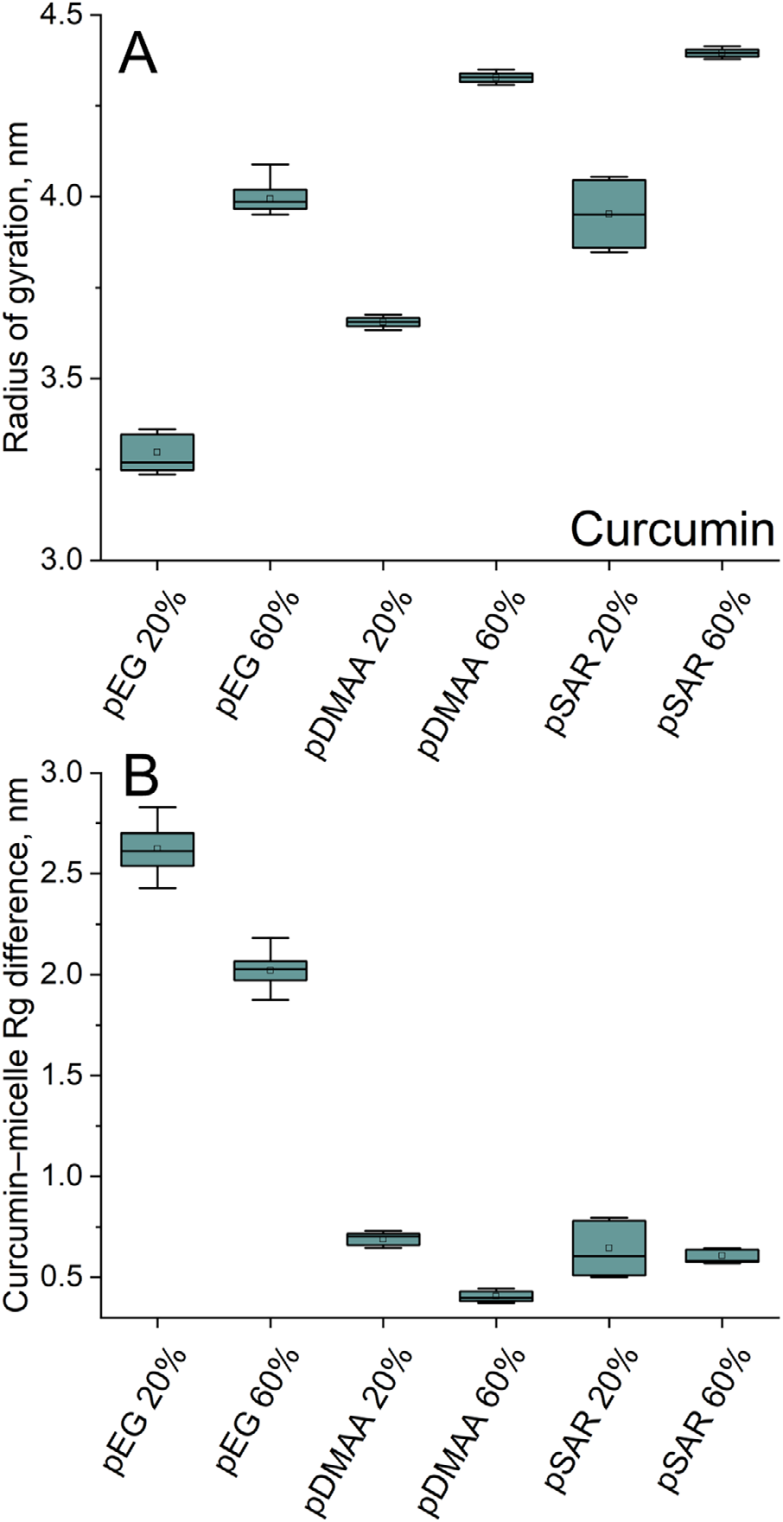
Boxplots showing (A) the $R_{g}$ of the curcumin atoms within pEG, pDMAA, and pSAR micelles, (B) difference between the $R_{g}$ of curcumin molecules and $R_{g}$ of the respective micelle.

We further characterized curcumin distribution by calculating the $R_{g}$ of curcumin molecules and comparing it to the micelle $R_{g}$ (Figure 8). In the pEG micelles, the curcumin $R_{g}$ was significantly smaller than in pSAR and pDMAA micelles at equivalent loading levels. The difference between micelle and curcumin $R_{g}$ was notably larger in pEG systems. This again indicates that curcumin is predominantly located within or near the hydrophobic B‐block when the corona is pEG. In contrast, pSAR and pDMAA micelles exhibited much smaller $R_{g}$ differences (<0.8 nm), suggesting that curcumin is more evenly distributed throughout the micelle and occupies nearly the entire internal volume. Based on the above, it can be suggested that further increases in drug loading beyond 60% in pSAR and pDMAA micelles would increase the micelle $R_{g}$ and expand sticky areas. Conversely, in pEG micelles, higher drug loading (if actually possible) would primarily result in filling the internal volume within the pEG corona, with displacement of water molecules; the thermodynamic feasibility of this scenario remains, however, unclear.

To complement the analysis of spatial distribution, we evaluated curcumin mobility by tracking their center of mass (COM) displacements. We quantified mobility as the percentage of frames in which COM remained within ±1 Å of its initial position (Figure S8). Clear differences emerged. pDMAA and pSAR micelles exhibited high curcumin retention, with 78% to 89% of COM remaining within ±1 Å of its initial positions (Table S6), indicating strong interactions and limited mobility, which could incidate stable encapsulation. In contrast, pEG micelles showed significantly higher curcumin mobility, particularly at 20% loading, with 54%–61% of COM within ±1 Å of its initial position. These findings indicate that pDMAA and pSAR micelles provide a more favorable environment for curcumin stabilization than pEG micelles. This enhanced stability reflects the higher internal density of pDMAA and pSAR micelles, which results from tighter curcumin packing and limited mobility. Conversely, the lower density of pEG micelles, resulting from its non‐compact A‐block structure, allows curcumin greater mobility and reduces its retention near initial positions.

### 3.4 Curcumin Cluster Within Polymeric Micelles

We further characterized micellar architecture by analyzing curcumin–curcumin interactions through cluster analysis. Representative curcumin cluster structures at the end of the simulation are shown in Figure S9. Within each micelle, the curcumin molecules preferentially aggregated into a primary cluster comprising most of the molecules. For the majority of micelles, a single cluster of curcumin molecules was identified. An exception occurred in pSAR micelles at 20% drug loading (replica #1, in total 198 curcumin molecules), where three distinct clusters were identified: the largest contained >90 curcumin molecules, while the two smaller clusters contained approximately 30 and 17 molecules, respectively.

The clusters remained stable throughout the entire production run with only occasional curcumin molecules at the outer surface detaching and reattaching. These findings confirm the COM analysis results and support the hypothesis from our previous study [41] that curcumin mobility occurs primarily during the initial equilibration stage.

We quantified the percentage of curcumin molecules incorporated into the largest cluster (Figure S10). At 20% drug loading, 95%–98% of the curcumin molecules in pEG micelles were in the largest cluster. In contrast, pSAR and pDMAA micelles contained approximately 75%–85% of the curcumin molecules in the largest cluster at the same loading. The higher clustering in pEG micelles reflects tighter encapsulation within the hydrophobic core, likely associated with stronger hydration of the PEG A‐block compared to pSAR and pDMAA micelles. At 60% drug loading, nearly all curcumin was incorporated into cluster structures across all micelle types.

**FIGURE 9:**
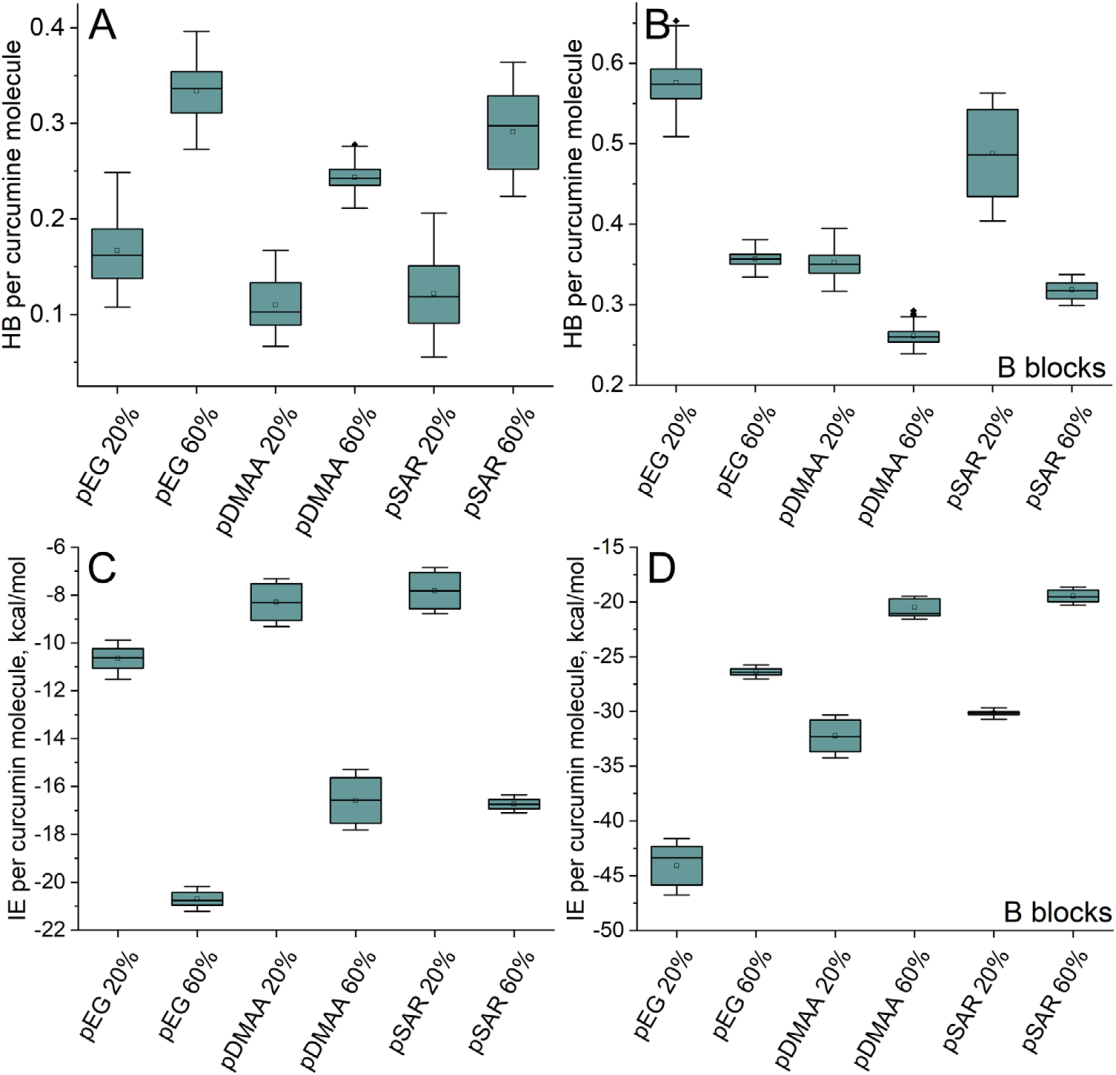
HB and interaction energy (IE) analysis. Normalized number of HBs per curcumin molecule between (A) curcumin molecules and (B) curcumin molecules and the polymer B‐blocks. Normalized interaction energies per curcumin molecule for (C) curcumin–curcumin and (D) curcumin‐B‐block interactions in pEG, pDMAA, and pSAR micelles.

Although most curcumin is part of the cluster, a substantial fraction remains in close proximity to the polymer B‐blocks (Figure 6). This finding indicates that the curcumin cluster is not densely packed but is rather penetrated by polymer chains. Consequently, curcumin interacts simultaneously with polymer chains and other curcumin molecules. To further characterize curcumin–curcumin and curcumin‐polymer interactions, we calculated the total number of HBs and the total interaction energy per curcumin molecule, enabling comparison between different micelles (Figure 9).

The curcumin–curcumin HB numbers were similar to the B‐block–curcumin HB numbers. At 60% drug loading, these values were approximately equal. For example, pEG micelles at 60% drug loading exhibited 0.33 HBs per curcumin for curcumin–curcumin interactions and 0.36 for B‐block–curcumin interactions. pDMAA micelles showed 0.24 and 0.26, respectively, while pSAR micelles showed 0.29 and 0.32, respectively, thus pDMAA shows significantly fewer hydrogen bonds. These findings indicate that curcumin–curcumin and B‐block–curcumin hydrogen bonding are comparable across all micelles at both drug loadings.

We performed a similar analysis of interaction energies per curcumin molecule (Figure 9). At 20% drug loading, curcumin–curcumin interactions were weaker than B‐block‐curcumin interactions. However, these interactions became increasingly comparable at higher drug loadings. For instance, in pEG micelles at 20% drug loading, curcumin–curcumin and curcumin‐B‐block interactions were –11 kcal/mol and –44 kcal/mol, respectively; at 60% loading, these values shifted to –21 kcal/mol and –27 kcal/mol, respectively.

Similar behavior was observed in pDMAA and pSAR micelles. At 60% loading, pDMAA micelles showed interaction energies of –20 kcal/mol for B‐block–curcumin and –17 kcal/mol for curcumin–curcumin. pSAR micelles showed –19 and –17 kcal/mol, respectively. These results demonstrate that at 60% drug loading, curcumin–curcumin and B‐block–curcumin interaction energies were nearly equivalent across all micelles.

Although the curcumin concentration increased, the normalized number of HBs between curcumin and the polymer B‐block remains comparable to that between curcumin molecules. In contrast, curcumin–curcumin interaction energy increased with concentration, while B‐block–curcumin interaction energy decreased. These opposing trends indicate that curcumin preferentially associates with other curcumin molecules at higher drug loadings.

## 4 Conclusions

In our search for alternatives to immunogenic pEG, we used all‐atom molecular dynamics (MD) simulations to investigate ABA triblock copolymer micelles with three different hydrophilic corona A‐blocks (pEG, pDMAA, and pSAR) and a common hydrophobic pBuOzi‐based B‐block core, in order to assess their influence on the encapsulation and behavior of the hydrophobic drug curcumin. Our findings demonstrate that A‐block chemistry and drug loading jointly determined critical micelle properties including size, hydration, drug distribution, and polymer‐drug interactions. Importantly, all three systems maintain structural stability at high drug loading (60% polymer/drug mass ratios). Experimental validation using complementary techniques (SAXS/SANS, cryo‐TEM, and fluorescence microscopy) and studies under physiologically relevant conditions will be essential to confirm these predictions and advance rational design of next‐generation delivery systems.

pEG micelles exhibited the largest radius of gyration and the highest hydration levels, confining curcumin predominantly to the hydrophobic core. In contrast, pDMAA‐ and pSAR‐based micelles moieties were more compact and less hydrated, with curcumin distributed uniformly throughout the micellar volume. Center‐of‐mass tracking showed reduced mobility of drug molecules in pDMAA and pSAR micelles. These results suggest that pDMAA and pSAR micelles provide more favorable conditions for the stabilization of curcumin molecules compared to the case of micelles with a pEG corona.

Cluster analyzes revealed that curcumin molecules form a single, stable cluster within each micelle. This cluster is not a densely packed aggregate but rather interpenetrated by polymer chains. Consequently, curcumin molecules simultaneously interact with both the polymer chains and other curcumin molecules. At 20% drug loading, curcumin–curcumin interactions are dominated by the polymer B‐block, whereas at 60% curcumin–curcumin and curcumin‐polymer interactions become comparable in magnitude.

The picture that emerges is one of a set of design trade‐offs that can be adjusted to achieve an optimal balance. pEG exhibits high hydration but low cargo affinity, pDMAA shows moderate hydration and high cargo affinity, and pSAR displays both moderate hydration and affinity. In our previous work  [41] we saw that pMeOx has hydration and affinity properties similar to pSAR whereas pEtOx shows low hydration and high affinity. Thus, moving forward, in designing new A‐block polymers for polymer coronas, trying to find polymers that optimally balance these two competing criteria for desired performance should be one of the design strategies pursued.

Hydrogen‐bonding analysis showed that the number of HBs between curcumin and the hydrophilic A‐blocks decreases in the order pDMAA > pSAR > pEG. In pEG micelles, only 0.3%–2% of A‐block oxygen atoms participate in H‐bonds with curcumin, compared to 9%–10% in pDMAA micelles.

Curcumin atoms at the micelle surface form distinct hydrophobic “sticky” patches that expand with increasing 60% drug loading. These sticky patches may have dual consequences: (1) they could compromise colloidal stability and enhance non‐specific binding to cell membranes and proteins, potentially altering pharmacokinetics, and (2) alternatively, they could facilitate drug release in response to environmental triggers.

It is important to note that MD simulations have inherent limitations, particularly in terms of feasible timescales and system sizes. Micellar structures and drug behaviors may depend on initial configurations, and thus further studies employing enhanced sampling techniques and experimental validation are necessary to verify the generality of our findings.

## Conflicts of Interest

The authors declare no conflicts of interest.

## Supporting information


**Supporting File**: smll74919‐sup‐0001‐SuppMat.pdf.

## Data Availability

Data and parameters are openly available at https://10.5281/zenodo.17962650.
