# Supplementary material for: Think Beyond The Core: Computationally Decoding the Hydrophilic Corona of Drug‐Loaded Polymer Micelles
Source: Small. 2026 Aug 8;22(54):e74919. doi: 10.1002/smll.74919 (PMC13614293; doi:10.1002/smll.74919)
Supplement: Supplementary file 1 [file SMLL-22-e74919-s001.pdf]

Think Beyond the Core: Computationally Decoding the Hydrophilic Corona of Drug-Loaded Polymer Micelles

Maksym Karachevtsev,*,†,‡ Josef Kehrein,¶ Terttu Hukka,§ Alex Bunker,† Mikko Karttunen,‖,⊥ and Robert Luxenhofer‡

†*Drug Research Program, Division of Pharmaceutical Biosciences, Faculty of Pharmacy, University of Helsinki, Viikinkaari 5 E, P.O. Box 56, 00014 University of Helsinki, Finland*

‡*Soft Matter Chemistry, Department of Chemistry, Faculty of Science, University of Helsinki, P.O. Box 55, 00014 University of Helsinki, Finland*

¶*Institute for Pharmacy and Food Chemistry, University of Würzburg, Am Hubland, 97074 Würzburg, Germany*

§*Tampere University, Faculty of Engineering and Natural Sciences, Materials Science and Environmental Engineering, Chemistry & Advanced Materials, P.O. Box 541, FI-33014 Tampere University, Finland*

‖*European Laboratory for Learning and Intelligent Systems (ELLIS) Institute Finland, Maarintie 8, 02150 Espoo, Finland*

⊥*Department of Technical Physics, University of Eastern Finland, P.O. Box 1627, FI-70211 Kuopio, Finland*

E-mail: maksym.karachevtsev@helsinki.fi

Table S1: List of all simulated systems, showing the polymer type and composition (20% or 60%), number of curcumin molecules, initial radius of gyration of the micelle polymer atoms (in nm), initial radius of gyration of the micelle B-block atoms (in nm), final box length (in nm), duration of the second equilibration step at 385 K, and the production run duration at 300 K for each replica.

System name	Number of curcumin molecules	Initial radius of gyration (Rg) of the micelle B-block atoms, nm	Initial radius of gyration (Rg) of the micelle, nm	Final box length, nm	Duration of 2nd equilibration (385 K)	Production run (300 K)
pEG 20% replica #1	170	3.4	11.1	26.5	2400 ns	400 ns
pEG 20% replica #2	170	3.7	11.2	26.9	2400 ns	400 ns
pEG 60% replica #1	510	5.1	11.7	27.2	2400 ns	400 ns
pEG 60% replica #2	510	4.7	11.6	28.4	2400 ns	400 ns
pDMAA 20% replica #1	180	4.5	6.3	15.7	1000 ns	400 ns
pDMAA 20% replica #2	180	4.5	6.4	16.7	1000 ns	400 ns
pDMAA 60% replica #1	540	4.6	6.3	17.5	1000 ns	400 ns
pDMAA 60% replica #2	540	4.8	6.5	15.8	1000 ns	400 ns
pSAR 20% replica #1	200	4.1	9.0	20.6	2000 ns	400 ns
pSAR 20% replica #2	200	4.1	9.0	20.7	2000 ns	400 ns
pSAR 60% replica #1	600	5.2	9.4	22.3	2000 ns	400 ns
pSAR 60% replica #2	600	5.6	9.8	23.2	2000 ns	400 ns

Table S2: Asphericities (Q) of micelles.

System name	Q (replica #1)	Q (replica #2)
pEG 20%	0.009	0.008
pEG 60%	0.013	0.016
pDMAA 20%	0.015	0.009
pDMAA 60%	0.015	0.015
pSAR 20%	0.013	0.036
pSAR 60%	0.024	0.019

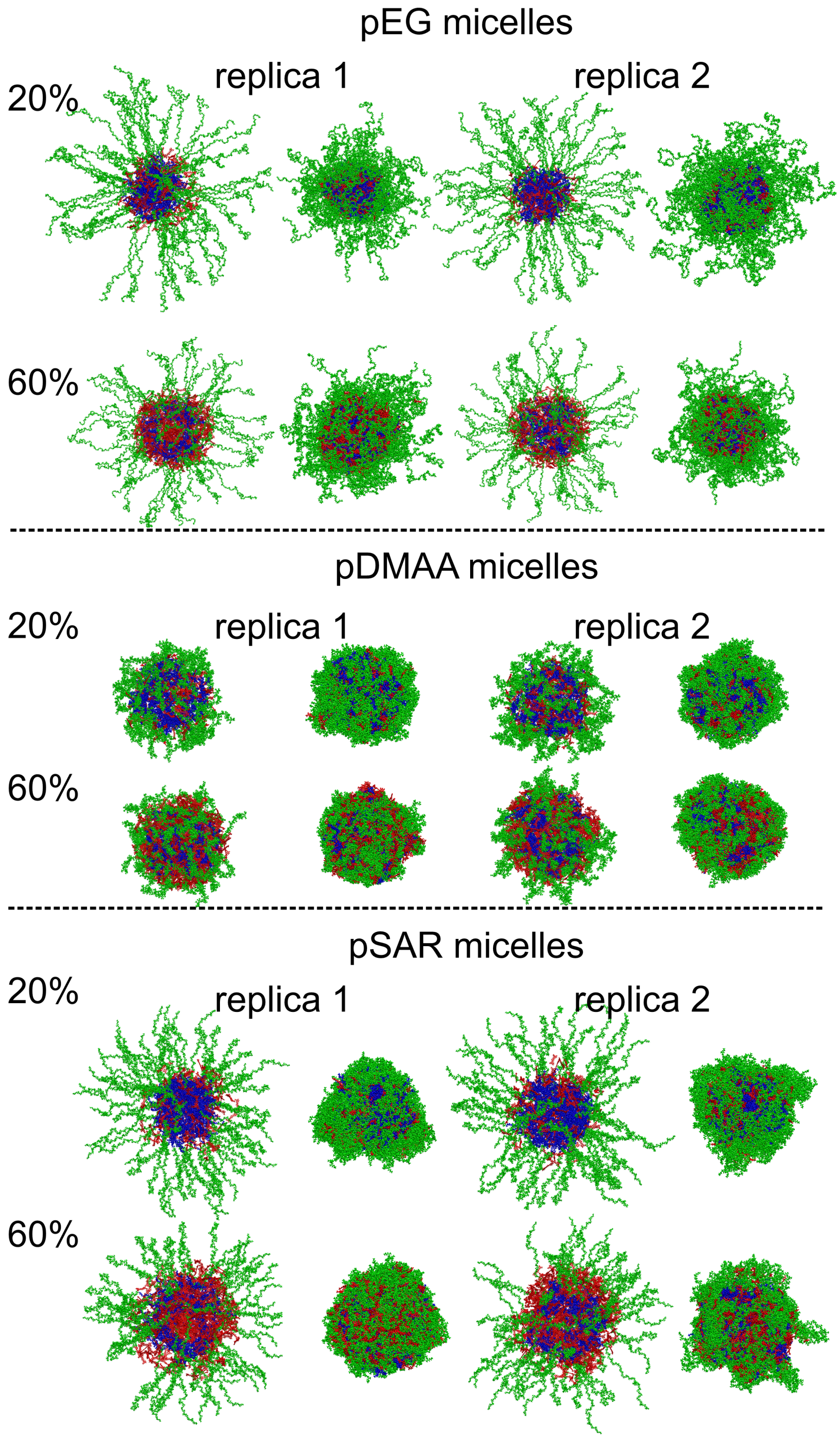

Figure S1: Initial and equilibrated micelles structures. Curcumin is shown in red, the hydrophilic A-blocks in green, and the hydrophobic B-blocks in blue. These structures represent the micelles after 400 ns of production runs.

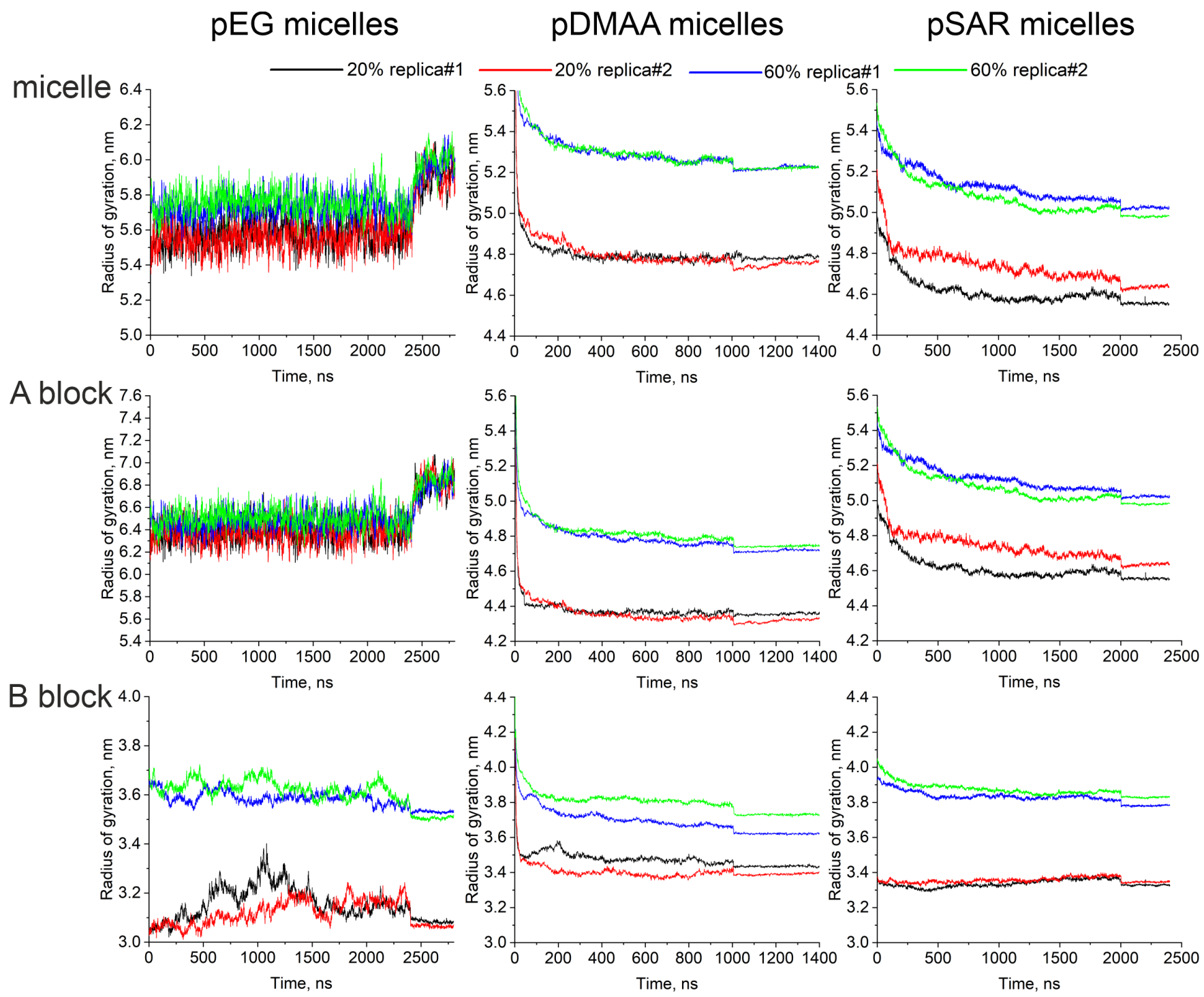

Figure S2: Time evolution of the radius of gyration (R_g) for pEG, pDMAA, and pSAR micelles. The top row shows the R_g of the entire micelle, while the middle and bottom rows correspond to R_g of the A- and B- blocks, respectively.

Table S3: Coordination numbers (N_c) of water oxygen atoms around A-blocks in pEG, pDMAA, and pSAR micelles at different drug loadings.

System name	Coordination number (N_c)
pEG 20%	1.53
pEG 60%	1.55
pDMAA 20%	1.17
pDMAA 60%	1.05
pSAR 20%	0.80
pSAR 60%	0.83

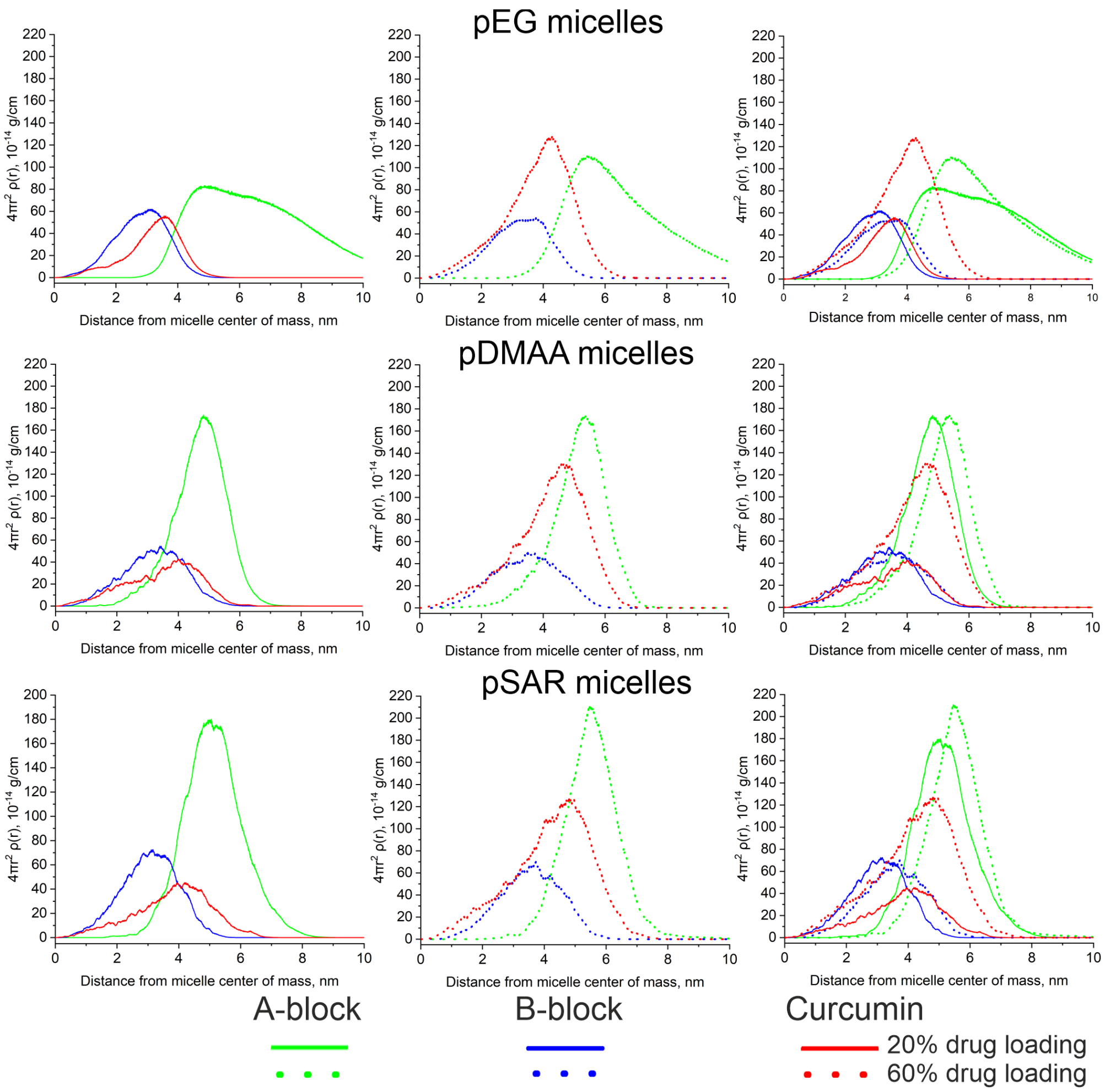

Figure S3: Radial mass distribution profiles of pEG, pDMAA, and pSAR micelles, averaged over two replicas. For each system, the distributions of the A-block (green), B-block (blue), and curcumin (red) are shown as a function of distance from the micelle center of mass. Solid lines correspond to 20% drug loading, and dotted lines to 60% drug loading.

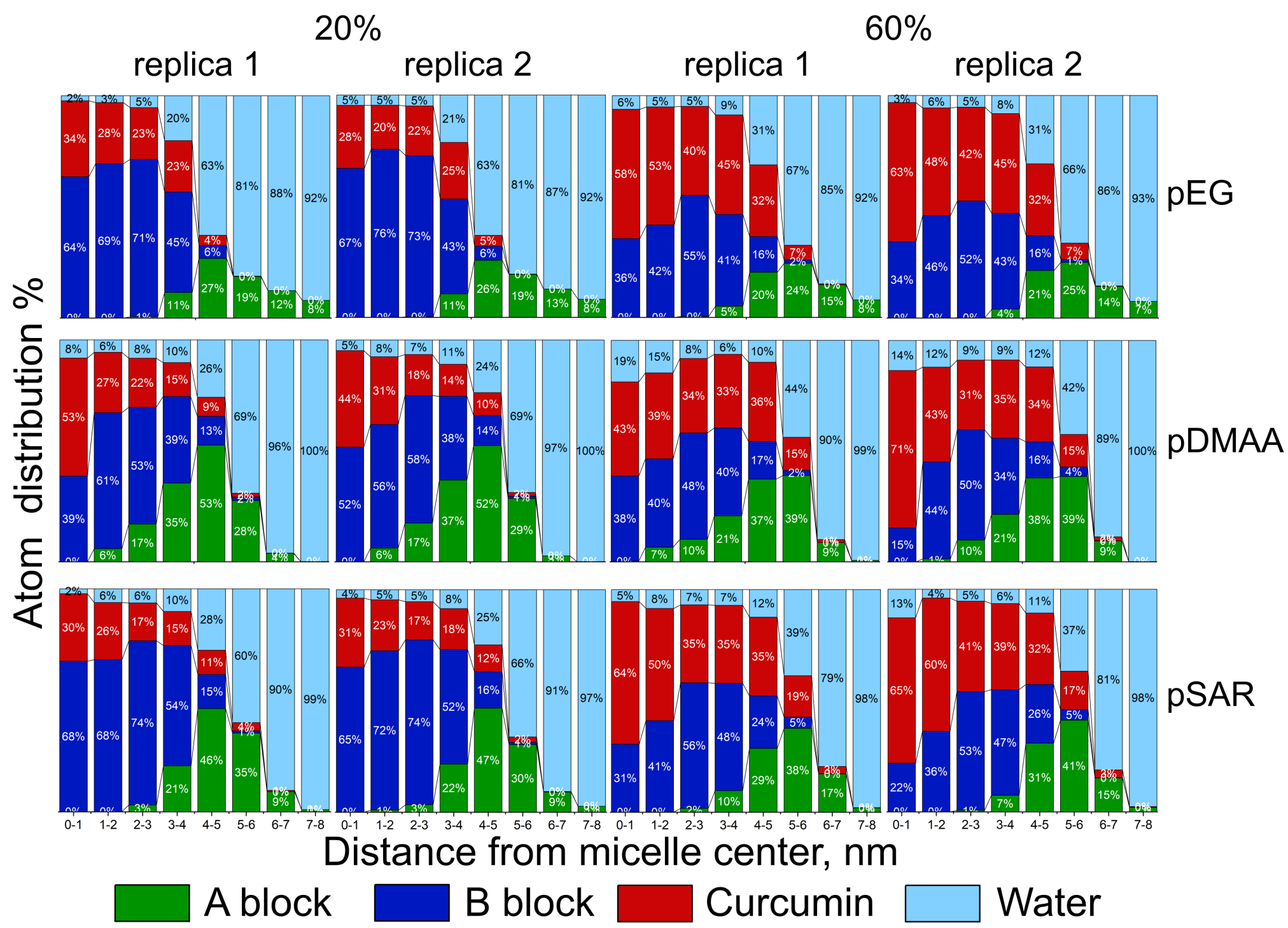

Figure S4: Final atomic density profiles (1 nm bin size) measured from the center of the micelle (geometric center of the B-block heavy atoms).

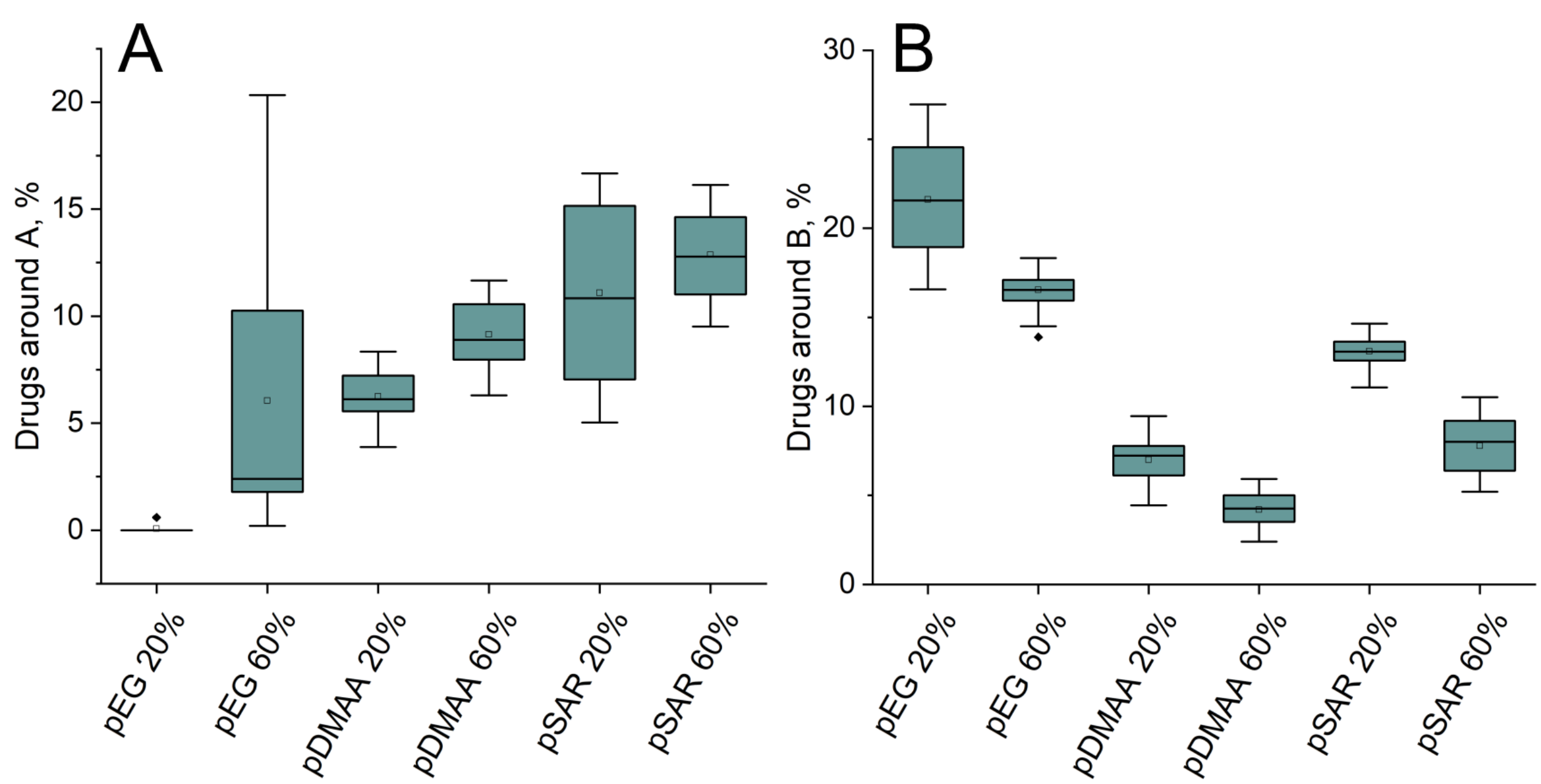

Figure S5: Number of drug molecules located within 0.5 nm of (A) A-block atoms and (B) B-block atoms, excluding molecules simultaneously located near both blocks.

Table S4: Parameters from bi-exponential fitting of the time autocorrelation functions of hydrogen bond lifetimes between micelle A- and B-blocks and curcumin, with the resulting characteristic hydrogen bond lifetimes τ_{HB}.

**A-block**					
System name	a	τ_1	b	τ_2	τ_{HB}
pEG 20% replica #1	0.145	58.12	0.849	1018.20	872.88
pEG 20% replica #2	0.172	56.20	0.822	729.11	608.99
pEG 60% replica #1	0.170	59.75	0.823	921.07	768.20
pEG 60% replica #2	0.158	59.07	0.835	840.50	711.15
pDMAA 20% replica #1	0.046	74.85	0.951	3625.66	3451.45
pDMAA 20% replica #2	0.056	66.26	0.940	3658.66	3442.85
pDMAA 60% replica #1	0.052	62.17	0.946	4041.89	3826.86
pDMAA 60% replica #2	0.077	74.57	0.920	4145.01	3819.15
pSAR 20% replica #1	0.228	81.43	0.767	592.35	472.90
pSAR 20% replica #2	0.136	77.13	0.860	1327.09	652.98
pSAR 60% replica #1	0.204	82.69	0.791	804.18	872.88
pSAR 60% replica #2	0.257	76.50	0.736	664.06	508.41
**B-block**					
pEG 20% replica #1	0.046	63.26	0.952	4186.70	3988.65
pEG 20% replica #2	0.043	56.78	0.955	4348.65	155.40
pEG 60% replica #1	0.050	54.97	0.948	4954.65	4699.76
pEG 60% replica #2	0.062	92.28	0.935	3569.28	3343.00
pDMAA 20% replica #1	0.048	75.24	0.950	4339.36	4126.00
pDMAA 20% replica #2	0.047	78.94	0.951	4682.16	4456.44
pDMAA 60% replica #1	0.046	67.28	0.952	6125.03	5834.12
pDMAA 60% replica #2	0.063	89.99	0.934	4341.54	4060.67
pSAR 20% replica #1	0.071	91.23	0.927	4231.26	3928.86
pSAR 20% replica #2	0.039	96.18	0.960	5277.98	5070.61
pSAR 60% replica #1	0.055	65.83	0.943	5372.86	5070.23
pSAR 60% replica #2	0.045	49.78	0.953	6597.69	6289.84

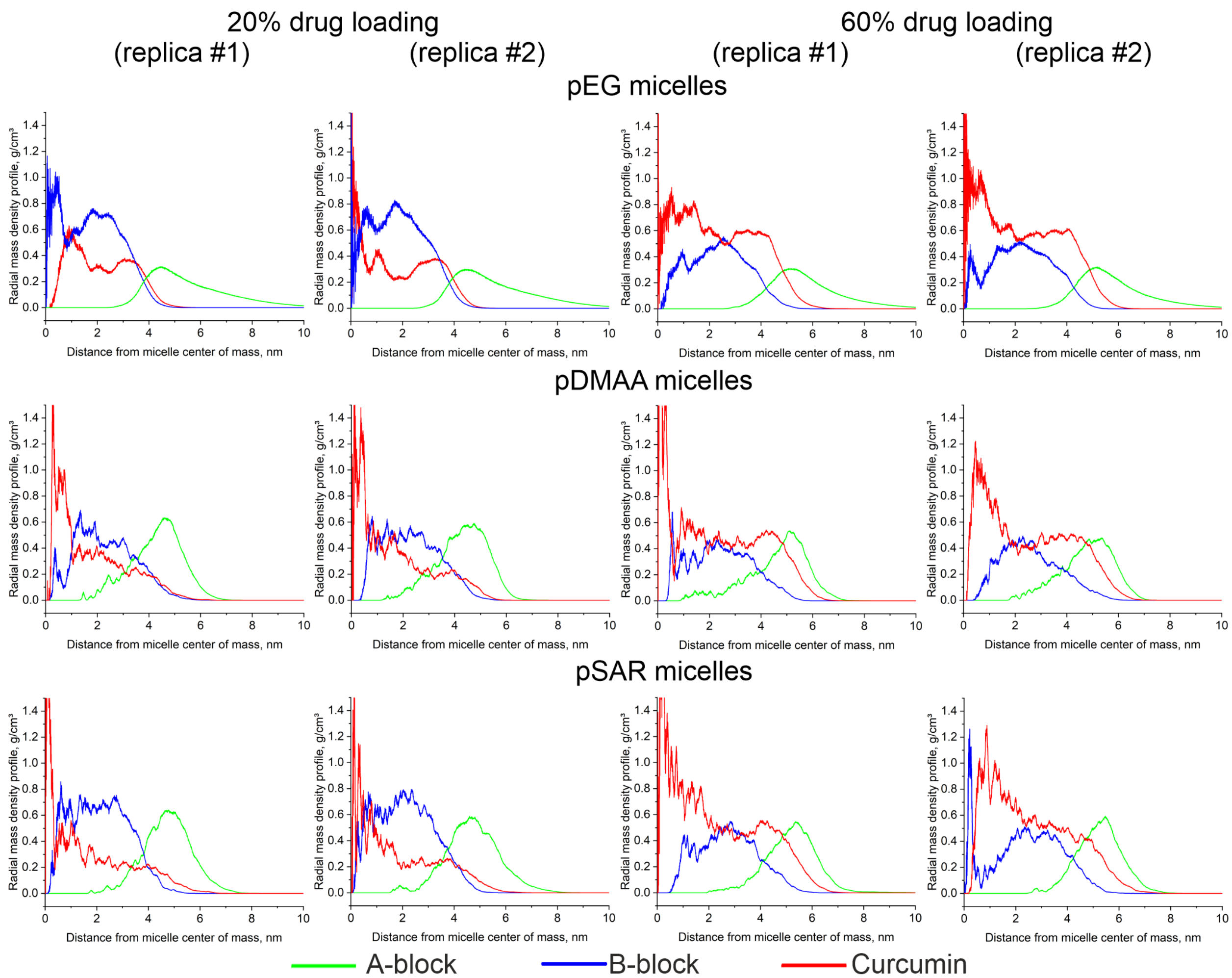

Figure S6: Radial mass density profiles of curcumin (red) and micelle polymer components (A-block - green, B-block - blue) as function of distance from the micelle center of mass for pEG, pDMAA, and pSAR micelles at 20% and 60% drug loading. Two independent replicas (replica #1 and replica #2) are shown for each system. The discrepancy between the two replicas in the limit of low distance is observed for the PEG micelles at 20% drug loading and results from poor statistics in this region due to the decreased volume of the counting shell.

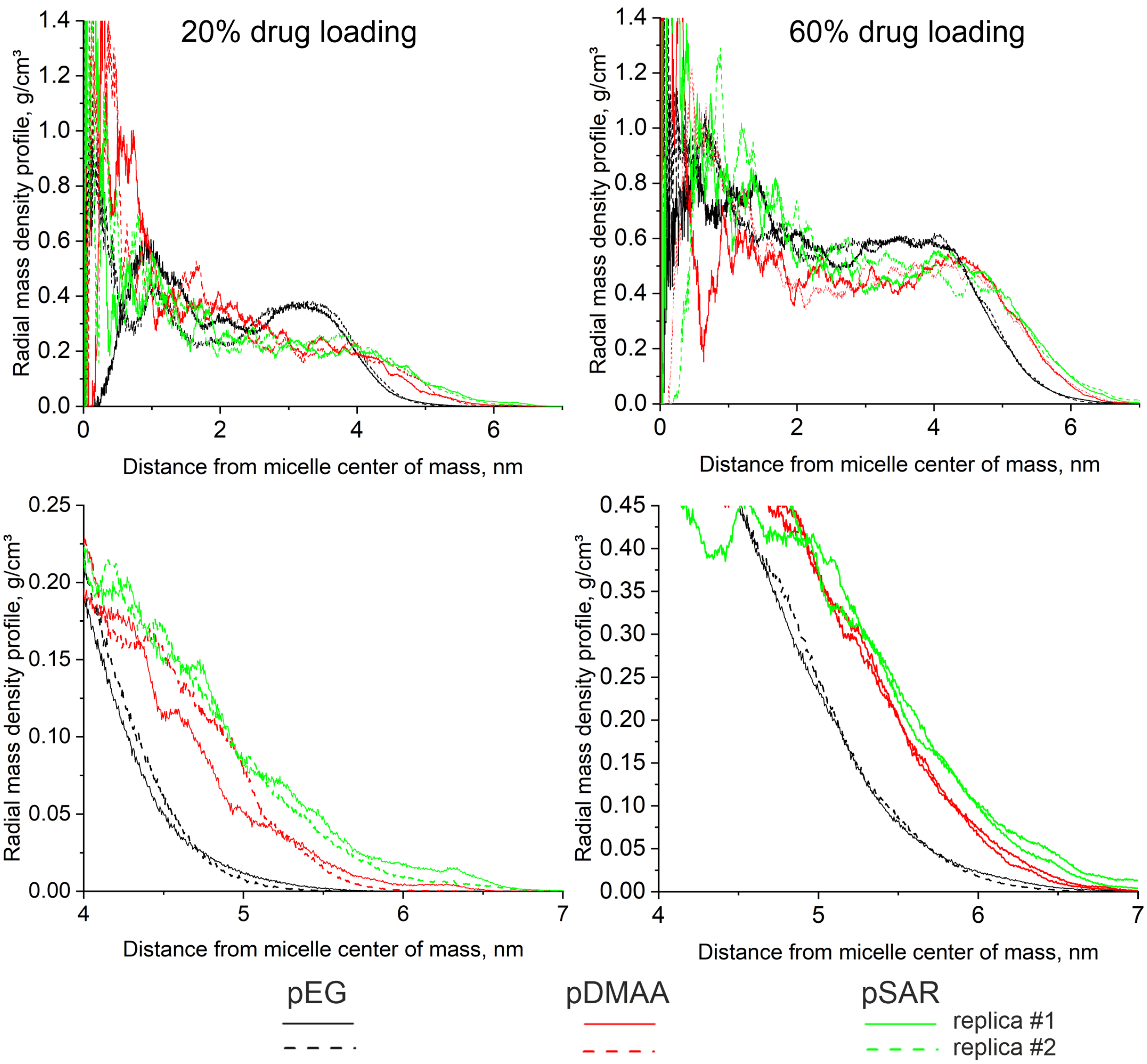

Figure S7: Radial mass density profiles of curcumin (red) and micelle polymer (black) as a function of relative distance from the micelle center of mass for pEG, pDMAA, and pSAR micelles at 20% and 60% drug loading.

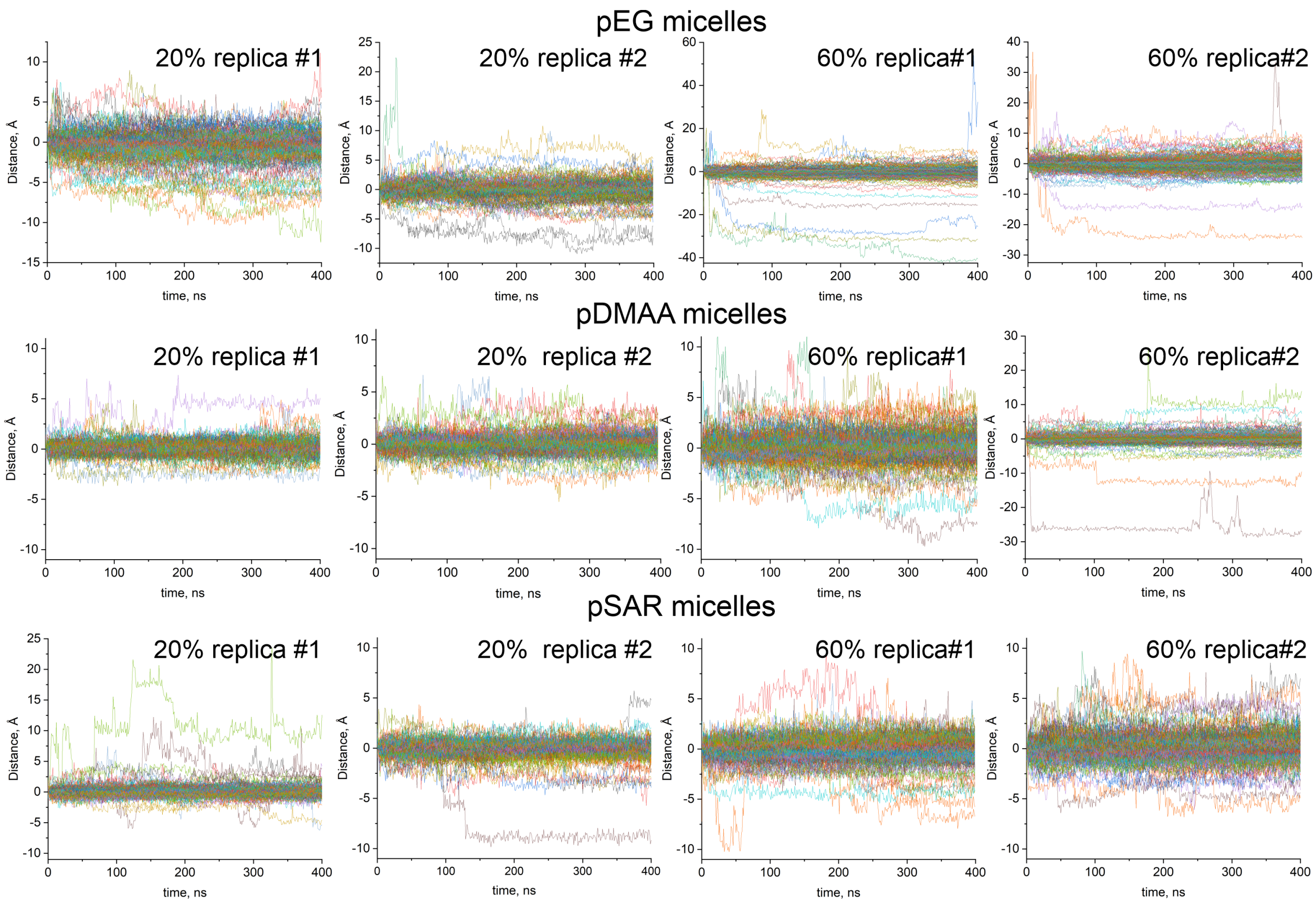

Figure S8: Time evolution of the center of mass (COM) displacements of curcumin molecules in pEG, pDMAA, and pSAR micelles at 20% and 60% drug loading. Each line represents the COM distance (in Å) of an individual curcumin molecule from its initial position over the production run.

Table S5: Number and surface localization of curcumin atoms in micelle surface/water shell region: absolute counts, fraction of total curcumin, and fraction of total surface atoms.

System name	Number of curcumin atoms on the surface of the micelle	Percentage of curcumin atoms on the micelle surface to the total number of curcumin atoms, %	Percentage of curcumin atoms on the micelle surface relative to the total number of surface atoms, %
pEG 20% replica #1	851	10.84	2.57
pEG 20% replica #2	894	11.26	2.74
pEG 60% replica #1	1959	8.39	6.31
pEG 60% replica #2	1880	7.98	6.18
pDMAA 20% replica #1	438	5.18	4.40
pDMAA 20% replica #2	498	5.89	5.01
pDMAA 60% replica #1	2435	9.60	22.12
pDMAA 60% replica #2	2179	8.59	20.14
pSAR 20% replica #1	721	7.75	6.48
pSAR 20% replica #2	482	5.15	2.86
pSAR 60% replica #1	1984	7.09	16.11
pSAR 60% replica #2	2226	7.91	18.15

Table S6: Stability of curcumin encapsulation: Percentage of frames with COM shifts within ± 1 Å for different polymer micelles.

System name	Percentage of COM shifts of curcumin molecules within ± 1 Å
pEG 20% replica #1	53.77
pEG 20% replica #2	60.93
pEG 60% replica #1	63.55
pEG 60% replica #2	66.38
pDMAA 20% replica #1	89.11
pDMAA 20% replica #2	83.76
pDMAA 60% replica #1	85.88
pDMAA 60% replica #2	78.52
pSAR 20% replica #1	85.08
pSAR 20% replica #2	88.15
pSAR 60% replica #1	88.49
pSAR 60% replica #2	87.27

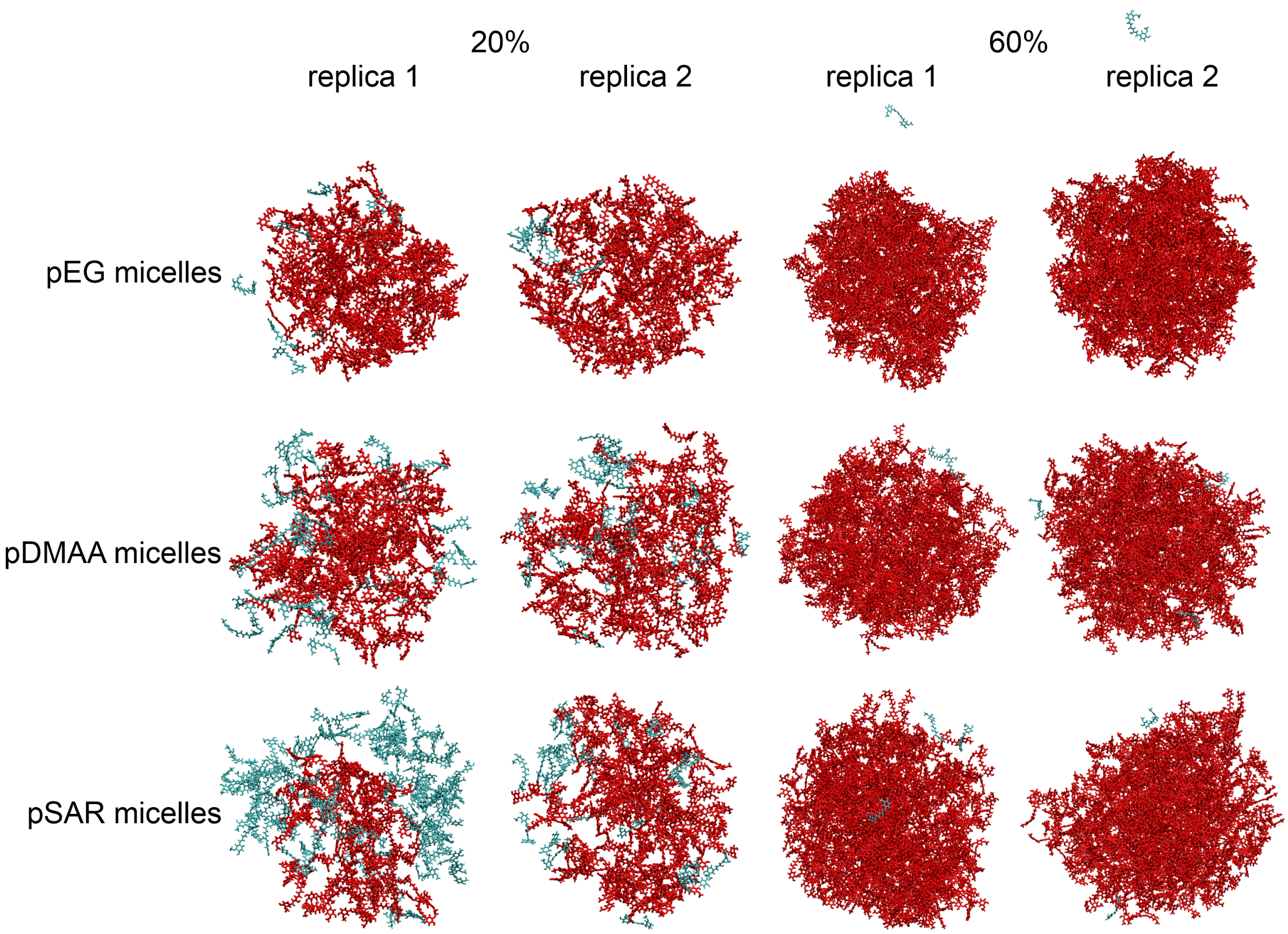

Figure S9: Curcumin cluster structures at the end of the simulation (polymers not shown). Curcumin molecules belonging to the largest cluster are shown in red, while those outside the largest cluster are shown in cyan.

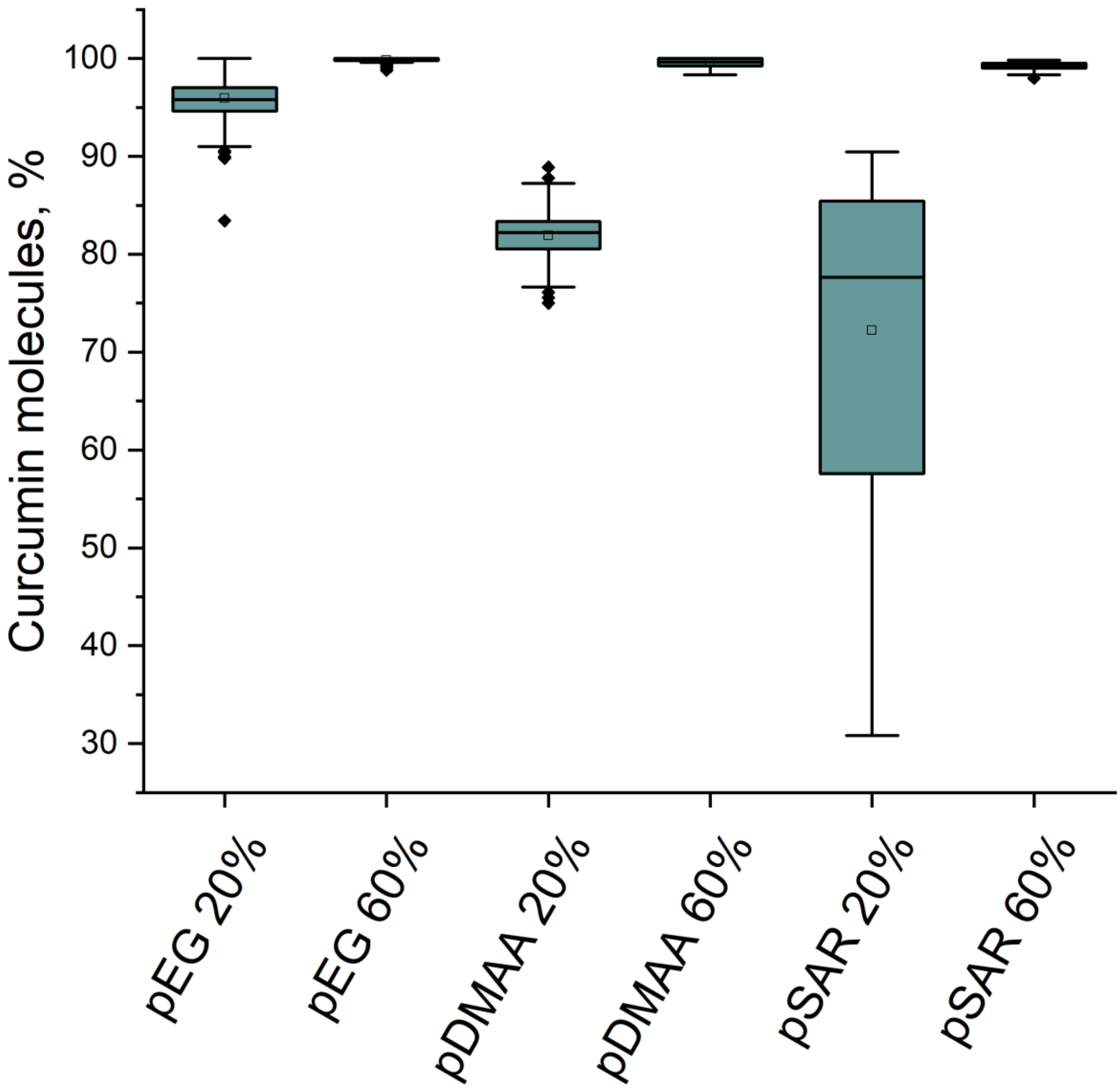

Figure S10: Percentage of curcumin molecules forming the largest cluster in PEG, pDMAA, and pSAR micelles at 20% and 60% drug loading.
